# Intracellular signalling and intercellular coupling coordinate heterogeneous contractile events to facilitate tissue folding

**DOI:** 10.1038/ncomms8161

**Published:** 2015-05-26

**Authors:** Shicong Xie, Adam C. Martin

**Affiliations:** 1 Computational and Systems Biology Program, Massachusetts Institute of Technology, Cambridge, Massachusetts 02142, USA; 2 Department of Biology, Massachusetts Institute of Technology, Cambridge, Massachusetts 02142, USA

## Abstract

Cellular forces generated in the apical domain of epithelial cells reshape tissues. Recent studies highlighted an important role for dynamic actomyosin contractions, called pulses, that change cell and tissue shape. Net cell shape change depends on whether cell shape is stabilized, or ratcheted, between pulses. Whether there are different classes of contractile pulses in wild-type embryos and how pulses are spatiotemporally coordinated is unknown. Here we develop a computational framework to identify and classify pulses and determine how pulses are coordinated during invagination of the *Drosophila* ventral furrow. We demonstrate biased transitions in pulse behaviour, where weak or unratcheted pulses transition to ratcheted pulses. The transcription factor Twist directs this transition, with cells in Twist-depleted embryos exhibiting abnormal reversed transitions in pulse behaviour. We demonstrate that ratcheted pulses have higher probability of having neighbouring contractions, and that ratcheting of pulses prevents competition between neighbouring contractions, allowing collective behaviour.

## Abstract (web summary)

Epithelial sheet migration proceeds via a series of actomyosin contractions, called pulses, that are stabilized, or ratcheted. Here, Xie and Martin develop a computational framework to determine how pulses are coordinated, and show that ratcheting of pulses allows collective migration by preventing competition with neighbouring pulses.

## Graphical abstract

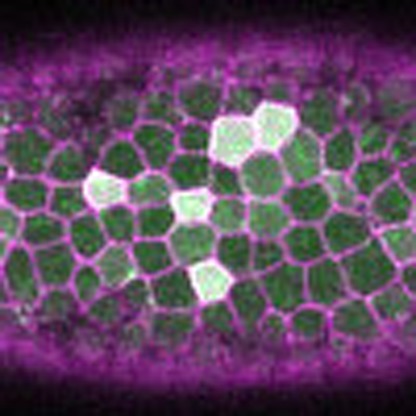

Tissue morphogenesis results from forces generated by myosin II (myosin) motors that contract networks of actin filaments (F-actin)^1^,^2^, often occurring in discrete force-generating events, called pulses or pulsed contractions, that acutely change cell shape^3^,^4^. One model system for studying tissue morphogenesis is *Drosophila* gastrulation, where the collective apical constriction of the presumptive mesoderm drives the folding of the embryo along its ventral side (ventral furrow)^5^,^6^. Pulsed contractions of the actin–myosin cortex drive apical constriction in individual cells; these contraction events can be stabilized in a ratchet-like manner, a process dependent on high expression of the transcription factor Twist (Twi)^7^. In order for the overall tissue to fold effectively, ventral furrow cells must collectively apically constrict. A widespread model of collective cell constriction is that a subset of cells initiates constriction, which triggers a population-level constriction^5^,^8^,^9^,^10^,^11^. Given that cell shape change can occur in steps, it is unclear whether changes in the properties of dynamic cell events can promote the transition to collective cell constriction. A systematic and quantitative characterization of transient, dynamic cell behaviours is necessary to investigate how pulsed contractions are temporally organized to constrict the tissue, as well as how pulses are spatially coordinated between neighbouring cells.

Here we develop a quantitative approach to identify and classify thousands of contractile events, showing that during wild-type *Drosophila* gastrulation, cells exhibit three predominant classes of contractile events: unconstricting, unratcheted and ratcheted. We show that Twi expression drives the biased transition in individual cells from a weak and unratcheted contraction state into a strong and ratcheted state. We also find that neighbouring cells are more likely to contract next to ratcheted pulses, and that ratcheting prevents competition between neighbouring pulses. These results demonstrate that dynamic transitions in individual cell behaviour promotes the transition into collective cell behaviour in the ventral furrow. Quantitatively defining different classes of contractile events is important to understand how cells interact within a tissue. Contractile pulses are observed in a wide variety of morphogenetic events^2^,^3^,^12^, and our findings suggest that the dynamic engagement of a ratcheting mechanism is a key event in eliciting collective behaviour in these systems.

## Results

### Computational identification of contractile events

**Figure 1:**
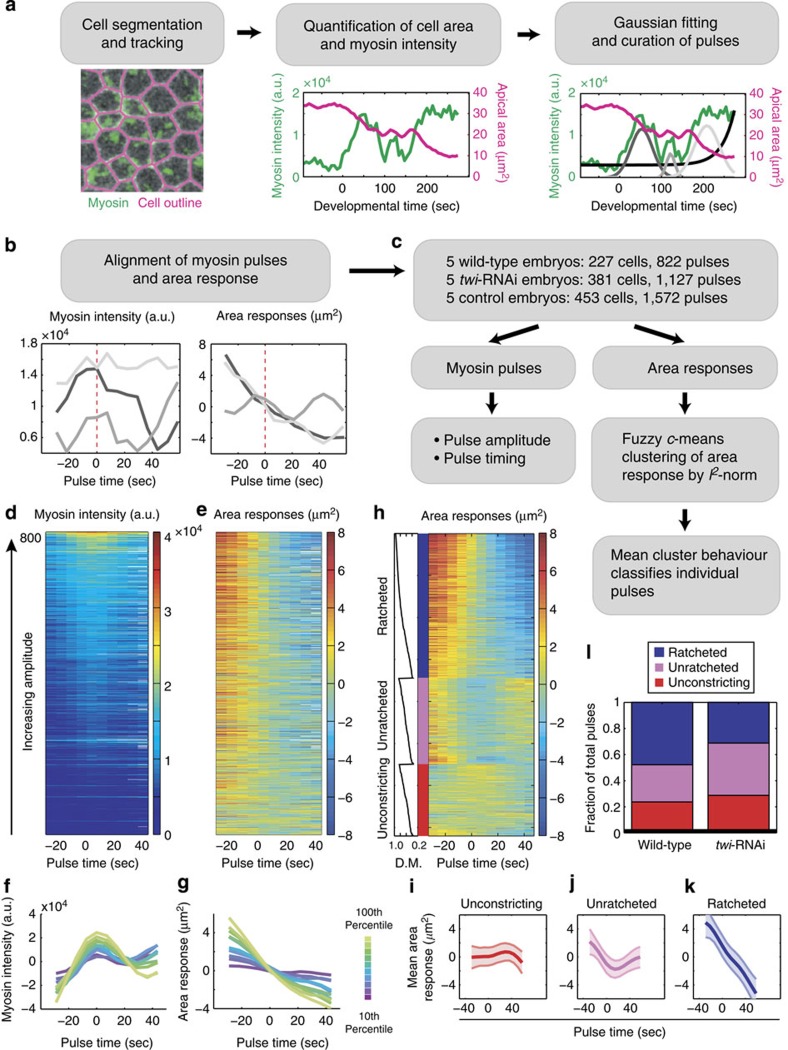
Computational framework for pulse identification, classification and quantification. (**a**) Cell outlines were segmented and tracked to measure the cell area (magenta) and apical myosin intensity (green)^41^. Myosin-intensity peaks (pulses) and background exponential increase (black) were identified by a multiple Gaussian fitting algorithm and subject to manual curation. The final curated Gaussian fits are shown in grey (right). (**b**) Pulses identified from the example cell in **a** were aligned by their centres (dotted line) to simultaneously quantify myosin intensity and area response. (**c**) Pipeline for quantifying myosin properties and area responses of individual pulses. Pulse magnitude and timing were measured by the Gaussian amplitude and mean, respectively. Local area response was clustered into behaviour classes by FCM and classified on the basis of the mean class behaviour. (**d**,**e**) Heatmaps of wild-type pulses identified and used in this study. Myosin intensity (**d**) and area response (**e**) of all pulses (*n*=822) identified from wild-type embryos (*n*=5) and sorted by pulse intensity. (**f**,**g**) Magnitude of apical constriction depends on the amplitude of myosin pulses. Average mean-centred myosin intensity (**f**) and average mean-centred area response (**g**) of pulses in various amplitude-bins. Colours denote the percentile-ranking in pulse amplitude (*n*>80 for each colour). (**h**) Pulses were clustered into three categories according to their area response behaviours. Heatmap shows the area responses to pulses (*n*=720) clustered by FCM into ratcheted (blue), unratcheted (magenta) and unconstricting classes (red). Within each class, pulses were also sorted by the degree of membership (D.M.) of each area response within their respective category (*left*). Pulses with missing data points were not categorized and not shown (*n*=102). (**i**–**k**) Average area response within each behaviour class. (**i**) Unconstricting pulses display no or minimal constriction (*n*=171). (**j**) Unratcheted pulses display unstabilized constrictions (*n*=205). (**k**) Ratcheted pulses display stabilized constrictions (*n*=344). Shaded areas represent s.d. (**l**) Fraction of pulses with given behaviour in wild-type (*n*=5) and *twi*-RNAi embryos (*n*=5).

In the *Drosophila* ventral furrow, myosin pulses generate force by rapidly contracting the apical F-actin cortex of a cell^7^,^13^,^14^,^15^; in addition to the acute assembly and disassembly seen during a pulse, apical myosin also increases over time to form a supracellular meshwork^16^. However, contractility generated by the cortex might not always result in a productive deformation of the cell apex, for example, if the cortex is not coupled to the apical margin, if the deformation is not stabilized or if there are external forces that counteract the generated force^7^,^15^,^17^. While different types of contractile events during ventral furrow formation have been described qualitatively, understanding the basis for how these events elicit tissue shape change requires a quantitative approach for classifying contractile events and assessing their coordination across the tissue. Using myosin intensity as a proxy for force generation, we developed a computational framework to identify contractile pulses, classify each pulse according to the behaviour of the resulting change in cell area and determine spatiotemporal relationships between pulses. We developed an iterative multi-Gaussian fitting approach to identify pulses from the myosin-intensity signal, using an exponential function to model the background increase in apical myosin levels (Fig. 1a, Supplementary Fig. 1, see Methods). To stringently verify this set of extracted pulses, we curated each putative pulse against a set of manually tracked pulses from the same embryo, yielding a set of validated myosin pulses from wild-type embryos (822 pulses, 277 cells, 5 embryos; Fig. 1a, Supplementary Fig. 2, see Methods). Our set of 822 pulses included all computationally and manually identified pulses that have been inspected and verified. The Gaussian parameters, mean and amplitude, were used to determine the timing of a pulse’s peak and its maximum myosin intensity, respectively (Fig. 1a,c). We aligned all identified pulses by their Gaussian means to visualize the mean-centred cell area response to a pulse (Fig. 1b). Sorting pulses by their amplitudes, we saw that the concomitant area responses also exhibited a gradient in the extent of constriction (Fig. 1d,e). Because raw intensities cannot be compared across individual movies, we binned pulses of the same percentile-ranking within their respective embryos, termed the amplitude-bin (see Methods). By averaging pulses within each amplitude-bin, we found a correlation between the rank of myosin pulses, and thus pulse magnitude, and the reduction in apical area (Fig. 1f,g). Taken together, these results show that there is a continuum of myosin pulse magnitudes within individual embryos during ventral furrow formation. Furthermore, we uncovered a dose-dependence of apical constriction on myosin pulse amplitude, which validates the use of myosin intensity as a proxy measurement for force generation.

### Three distinct classes of pulses during tissue invagination

**Figure 2:**
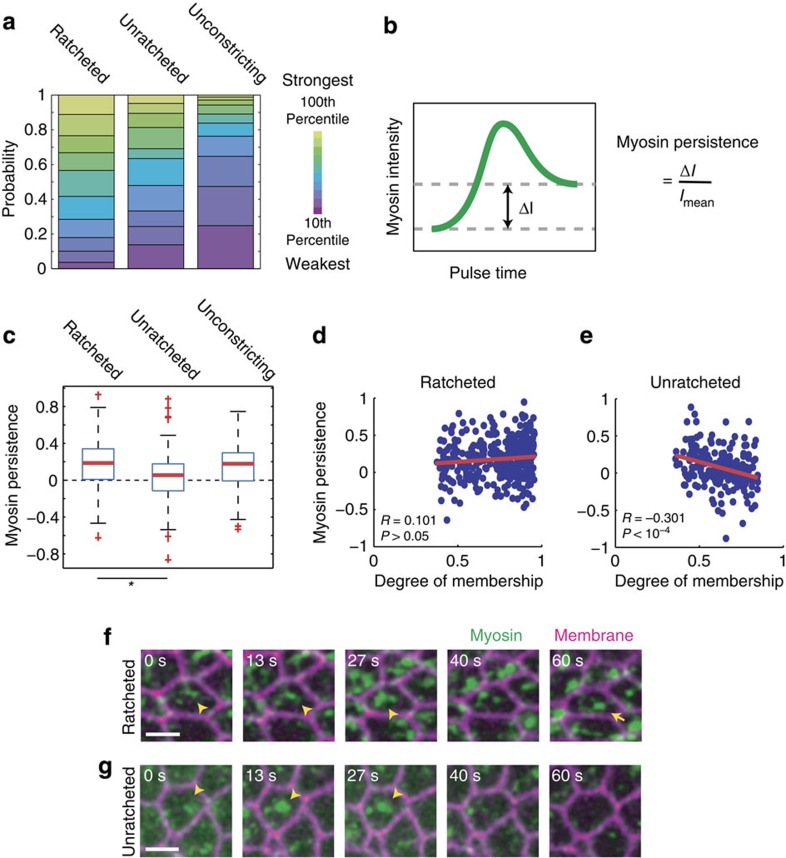
Ratcheting of contractile pulses correlates with persistent myosin structures. (**a**) Unconstricting pulses are enriched in low-amplitude myosin pulses. Distributions of pulse amplitude-bin for each pulse behaviour class are shown. (**b**) Schematic for the myosin persistence measurement. Myosin persistence is defined as the difference between the before-peak and after-peak myosin intensity minima (Δ*I*) normalized by the average intensity (*I*_mean_) during the pulse. (**c**) Ratcheted pulses display more persistent myosin after the pulse measured for wild-type cells. Myosin persistence measured for wild-type ratcheted and unratcheted pulses reveal the lack of persistent myosin intensity in unratcheted pulses (*P*<10^−7^, unpaired *t*-test). Red bars represent sample medians and boxes demarcate the 25th and 75th percentiles. (**d**,**e**) Myosin persistence is associated with pulse-ratcheting. The degree of membership in the ratcheted class (**d**) correlates with myosin persistence (*R*=0.101, *P*>0.05), while the degree of membership in the unratcheted class (**e**) significantly anticorrelates with myosin persistence (*R*=−0.301, *P*<10^−4^). Lines represent best-fit lines. (**f**,**g**) Representative images of a ratcheted pulse (**f**, arrowhead) show persistent myosin structures (**f**, arrow), while an unratcheted pulse (**g**, arrowhead) does not. Scale bars, 5 μm.

To determine whether wild-type embryos exhibit different classes of pulses that lead to different area responses, we used fuzzy *c*-means (FCM), an unsupervised clustering method, to group pulses with similar area response behaviours (Fig. 1c, see Methods)^18^,^19^. FCM clustering not only classifies pulses, but also quantitatively assesses the degree of membership of a pulse in a given class (Fig. 1h, degree of membership, *left*). We co-clustered the area responses of wild-type pulses alongside validated pulses from *twi*-RNAi embryos, which are known to exhibit unratcheted pulses (Supplementary Fig. 5a,b; 1,127 pulses, 381 cells, 5 embryos)^7^. Pulses clustered into three behaviour classes, which we termed ratcheted, unratcheted and unconstricting pulses (Fig. 1h). On average, ratcheted pulses exhibited apical constrictions that did not relax (Fig. 1k, Supplementary Fig. 3a). Unratcheted pulses also showed apical constriction; however, the constricted area was not stabilized and the apical domain subsequently expanded (Fig. 1j, Supplementary Fig. 3b). In contrast, unconstricting pulses did not display measurable apical constriction (Fig. 1i, Supplementary Fig. 3c). To validate the use of three clusters, we used principal component analysis (PCA) to show that three principal components captured >90% of the total variance (Supplementary Fig. 4a,c). In addition, PCA reveals that these clusters do not represent discrete, well-separated clusters but rather different parts of a continuum of dynamic cell behaviours (Supplementary Fig. 4b,d, see Methods). Consistent with previous reports, we observed that ratcheted pulses comprise the largest fraction of wild-type pulse behaviour, while pulses in *twi*-RNAi embryos were enriched in the unratcheted behaviour (Fig. 1l)^7^. Next, we asked whether the unconstricting behaviour reflects different myosin pulse amplitudes. The unconstricting pulses could represent contractile events in which the apical actin–myosin meshwork is not mechanically coupled to junctions at the apical margin^15^; however, our quantitative analysis of pulse amplitudes demonstrates that unconstricting pulses are significantly enriched in pulses of the lowest ranked amplitude-bins (Fig. 2a), suggesting the alternative possibility that this class represents weak pulses that fail to change cell shape. Both ratcheted and unratcheted pulses have a significantly higher probability of being higher amplitude pulses than unconstricting pulses (Fig. 2a).

### Ratcheted constrictions have higher myosin persistence

Previous models of the ratchet mechanism posited that ratcheting is caused by myosin structures persisting after a contractile pulse; however, we did not previously have a quantitative metric of ratchet engagement^16^,^20^. To test our model, we measured myosin persistence for each pulse, defined as the normalized difference between the minimal myosin intensity before and after the pulse peak (Fig. 2b). Supporting our model, we found that myosin was more persistent in ratcheted pulses compared with unratcheted pulses; furthermore, unratcheted pulses had average persistence close to zero, suggesting a lack of residual myosin structures after the dissipation of the pulse (Fig. 2c,f,g). In addition, when we examined the degree of membership of pulses in a particular behaviour class as given by FCM, we saw that membership in the unratcheted class anticorrelates with myosin persistence, demonstrating that the most unratcheted constrictions tend to lack myosin persistence (Fig. 2e). The correlation between membership in the ratcheted class and myosin persistence was positive, although not significant (Fig. 2d). These results support a ratchet mechanism in which persistent apical myosin structures help to stabilize pulsed contractions. In addition, we show that lower Twi levels increase the prevalence of an unratcheted behaviour already present in the wild-type system.

### Ventral furrow cells constrict as a single population

**Figure 3:**
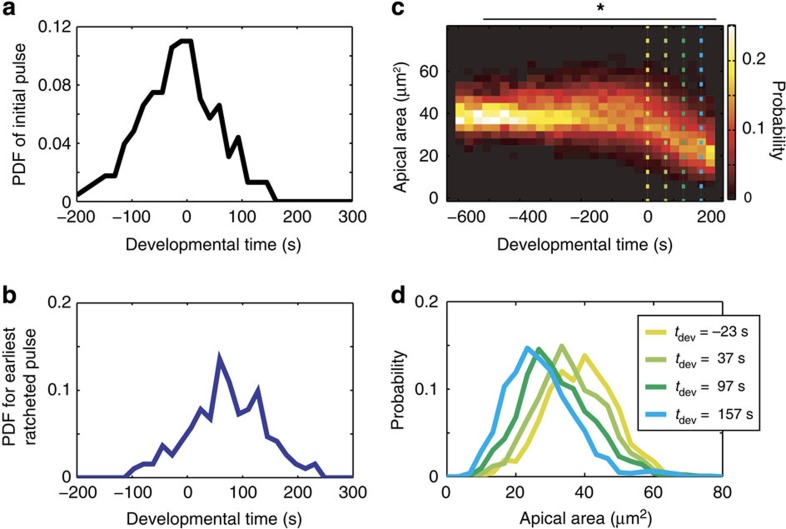
Ventral furrow cells initiate contraction as a single population. (**a**) Distribution of timing of the initial pulse is unimodal. The timing of the first pulse from each wild-type cell is quantified and exhibits a single mode in density. The probability density function (PDF) is shown. (**b**) Distribution of timing of the first ratcheted pulse is unimodal. The timing of the first pulse from each wild-type cell that was ratcheted is quantified. (**c**) Apical area decreases smoothly as a single population. The probability distributions of apical areas is quantified across temporal bins in developmental time and shows that cells constrict as a single population. Dotted lines refer to plots in **d**. *P>0.1, Hartigan’s *dip* test for nonunimodality^21^. (**d**) The distribution of apical area is shown for four time points during ventral furrow formation. Colours correspond to dotted lines in **c**.

We next investigated how collective apical constriction occurs in the ventral furrow. Previous research, mostly on fixed embryos, resulted in a model that a subpopulation of cells initiates constriction, termed the stochastic phase of constriction, which triggers the coherent constriction of the remaining cells, resulting in tissue contraction and folding^5^,^8^,^9^. We examined whether we could detect cell populations with distinct contractile dynamics. To compare the timing of ventral furrow formation across wild-type embryos, we temporally aligned movies using the onset of net tissue contraction as a reference point, set to *t*=0 (Supplementary Fig. 6a). We quantified the timing of pulse initiation in wild-type cells and found that it exhibits a unimodal distribution in time (Fig. 3a; *P*>0.9 Hartigan’s *dip*-test for nonunimodality^21^). Similarly, the timing of the first ratcheted pulse is unimodal (Fig. 3b; *P*>0.8). In addition, cell apical areas change as a unimodal distribution in time (Fig. 3c,d; *P*>0.1), suggesting that there are not distinct subpopulations of cells that differentially initiate constriction.

### Cells transition from unratcheted to ratcheted pulses

**Figure 4:**
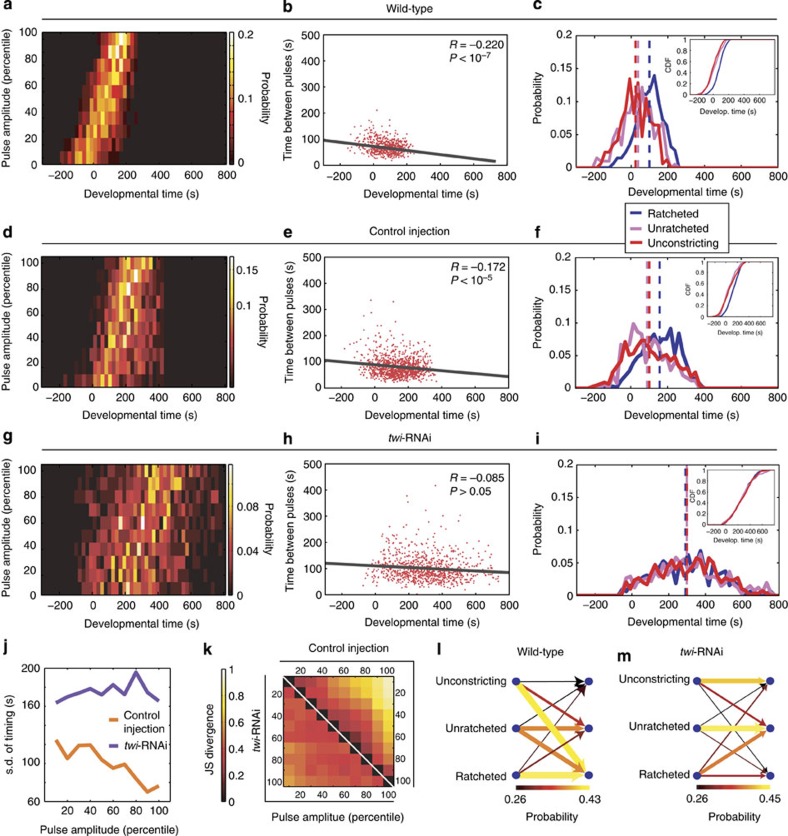
Twi expression is required for the temporal progression of contractile states. (**a**) Wild-type pulses progressively increase in magnitude. Probability density functions of the timing of pulses of increasing amplitude-bins are shown. (**b**) Wild-type pulses become increasingly frequent. The time interval between consecutive pulses is shown with respect to developmental time. Line shows best-fit. (**c**) Wild-type pulses transition from unconstricting and unratcheted pulses to ratcheted pulses. Probability density functions of the timing of different pulse behaviour classes are shown. (Inset) Cumulative distribution function. Dotted lines demarcate the respective mean timing. (**d**,**g**) *twi*-RNAi disrupts the temporal ordering from weak to strong pulses. Probability density functions of the timing (*x* axis) of pulses of different amplitude-bins (*y* axis) for five control (**d**) and five *twi*-RNAi embryos (**g**). (**e**,**h**) Period between consecutive pulses decreases more in control embryos (**e**) than in *twi*-RNAi embryos (**h**). Lines show best-fit lines. (**f**,**i**) Probability density functions of the timing of pulses of different behaviours show ratcheted pulses occurring after unratcheted and unconstricting pulses in control embryos (**f**), but co-occurring with them in *twi*-RNAi embryos (**i**). (Insets) Cumulative distribution functions. Dotted lines show the respective mean timing. (**j**) s.d.’s in pulse timing show that *twi-RNAi* pulses of similar amplitude-bin occur over a broader time period than corresponding control pulses. (**k**) *twi*-RNAi disrupts the temporal separation of pulses with different amplitudes. Jensen–Shannon divergence (see Methods) quantifies the dissimilarity of timing between pulses from different amplitude-bins. The lowest- and highest-amplitude pulses from control cells (upper right corner) display more divergent timing than those from *twi*-RNAi cells (lower left corner), reflecting greater separation and ordering in timings of pulses with different magnitudes in control compared with *twi-RNAi* tissues. (**l**,**m**) Twi expression promotes individual cells to transition into the ratcheted state. The probability of a cell transitioning from having a pulse of a given behaviour class (left columns) into having a subsequent pulse of another behaviour class (right columns) are shown. Wild-type cells (**l**) but not *twi*-RNAi cells (**m**) show biased transitions to the ratcheted state. Both the colours and the widths of the arrows represent the probability of transition.

We found that, while contractile pulses initiate before net tissue contraction, there is not a distinct subpopulation of ‘initiator’ cells. Therefore, we asked whether changes in the dynamic behaviour of individual cells could explain the onset of collective cell behaviour. We found that the amplitudes of myosin pulses increase progressively with respect to developmental time, with the weakest pulses occurring around or before *t*=0 and the strongest pulses occurring after *t*=0 (Fig. 4a). Furthermore, the time interval between consecutive pulses within a single cell displayed an anticorrelation with developmental time (*R*=−0.220, *P*<10^−7^). The period decreases by 8 s per minute from the average periodicity of 85 s seen before the onset of constriction, suggesting that in addition to increasing in amplitude, pulses also become more frequent (Fig. 4b). Finally, pulses become increasingly ratcheted over developmental time. Before *t*=0, most pulses are unconstricting or unratcheted (Fig. 4c), with only 15% of pulses being ratcheted (Supplementary Fig. 6d,e). In contrast, over 60% of pulses occurring after *t*=0 are ratcheted (Fig. 4c, Supplementary Movie 1). This transition in pulse behaviour occurs at the cell level, where individual cells are more likely to undergo transitions from unconstricting or unratcheted into ratcheted pulses than the reverse (Fig. 4l, Supplementary Fig. 6c). These results show that there is a temporal programme of increasing myosin pulse amplitude, frequency and ratcheting during ventral furrow formation, and that once in the ratcheted state, cells are more likely to continue to have ratcheted pulses.

### Twi expression promotes biased transitions in pulse class

We hypothesized that the kinetics of signalling events downstream of Twi expression may be required for cells to properly order contractile events during tissue folding. We examined *twi*-RNAi embryos and saw that the temporal coordination of pulses was disrupted in several aspects. First, pulses within individual embryos were no longer ordered over time from lower to higher amplitudes (Fig. 4d,g, Supplementary Fig. 7c–f). In control embryos, pulses within a single amplitude-bin occur on average within 70–120 s of each other, whereas in *twi*-RNAi embryos, pulses within the same amplitude-bin occur over a broader time distribution of 160–200 s (Fig. 4j). In addition, while lower magnitude control pulses on average precede higher ones, *twi*-RNAi pulses of different magnitudes are not as well separated in time. We used Jensen–Shannon divergence (JSD) to compare the timing of pulses of different amplitude-bins, where a value of zero denotes identical distributions and larger values denote increasing dissimilarity. We demonstrate that, whereas low- and high-amplitude pulses in control cells occur with dissimilar timing (Fig. 4k, upper right), those from *twi*-RNAi cells occur with much more similar timing (see Fig. 4k, lower left). The absolute magnitude of constriction rates is similar between Twi-depleted and control embryo pulses, suggesting that Twi does not simply alter the dynamic range of the magnitude of contractile pulses (compare Fig. 1e,g with Supplementary Fig. 5b,d). However, we cannot rule out that the absolute levels of force are different between embryos and that the dynamic range of contraction forces in *twi*-RNAi embryos is lower. Nevertheless, within individual embryos, *twi*-RNAi clearly disrupts the ordering of pulse amplitudes from weakest to strongest. Second, the temporal increase in pulse frequency is inhibited by *twi*-RNAi. Pulse period begins at an average value of 95 s, decreasing by 2 s per minute as opposed to the 8 s per minute seen in wild type (Fig. 4b,e,h). Finally, the tissue-level transition to predominantly ratcheted pulses is also abolished, with ratcheted, unratcheted and unconstricting pulses co-occurring in developmental time (Fig. 4f,i, Supplementary Fig. 7g,h, Supplementary Movie 2). This lack of transition to the ratcheted state occurred at the individual cell level, with cells having unratcheted pulses failing to proceed to ratcheted pulses and cells with ratcheted pulses abnormally reverting back to unratcheted pulses (Fig. 4l,m). Therefore, Twi expression promotes biased transitions between pulse classes, directing cells from weaker, less frequent and unratcheted pulses to stronger, more frequent and more ratcheted pulses.

### Twi promotes increased levels of stable medioapical Rok

**Figure 5:**
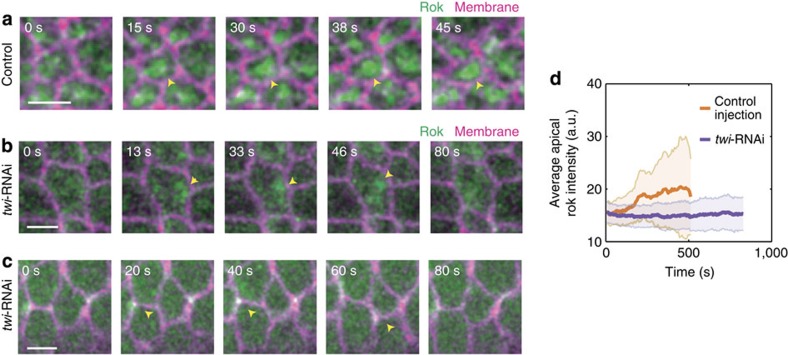
Twi expression promotes the medioapical stabilization of Rok. (**a**) Rok dynamically coalesces and forms a stable medioapical focus in control-injected cells (arrowhead). Scale bar, 5 μm. (**b**,**c**) *twi*-RNAi disrupts the medioapical stabilization of Rok. Rok still dynamically pulses medioapically (**b**, arrowhead) and junctionally (**c**, arrowhead) in *twi*-RNAi cells but does not form a stable medioapical focus. Scale bars, 5 μm. Rok images are maximum projections of the top 5-μm slices. (**d**) *twi*-RNAi disrupts the temporal increase in apical Rok localization. Average Rok intensity of apical Rok images from the same control-injected (**a**) and *twi*-RNAi cells (**b**). Error bars represent s.d.’s.

To elucidate the mechanism by which Twi expression temporally coordinates the transition in contractile state, we imaged the dynamics of Rho-kinase (Rok) in *twi*-RNAi cells. In the ventral furrow, Rok acts downstream of Twi expression to phosphorylate and activate myosin^13^,^14^,^22^. Previous studies showed that Rok exhibits apical pulses with spatiotemporal dynamics similar to those of myosin pulses, and eventually forms a stable, concentrated medioapical focus^13^,^14^. In *twi*-null embryos, Rok fails to form a medioapical focus and instead eventually localizes to tricellular junctions^13^. How depletion of Twi by RNAi, which results in clear contractile pulses and cell shape fluctuations, affects Rok localization is unknown. In control-injected embryos, we saw pulsatile Rok that progressively builds up into a stable medioapical focus, consistent with previous reports (Fig. 5a, arrowheads)^13^,^14^. In *twi*-RNAi cells, Rok is present in transient medioapical as well as junctional pulses, but never assembles into stable medioapical or junctional foci (Fig. 5b,c, arrowheads). Furthermore, the overall intensity of medioapical Rok is reduced in the *twi*-RNAi embryos and fails to increase compared with wild-type embryos (Fig. 5d). Therefore, the Twi-mediated increase in Rok medioapical localization and organization into a stable focus may be critical for the transition to intense and ratcheted myosin pulses, which could help to maintain persistent apical myosin and ensure that cells are in a contractile state where ratcheted pulses predominate.

### Ratcheting prevents competition between neighbouring pulses

**Figure 6:**
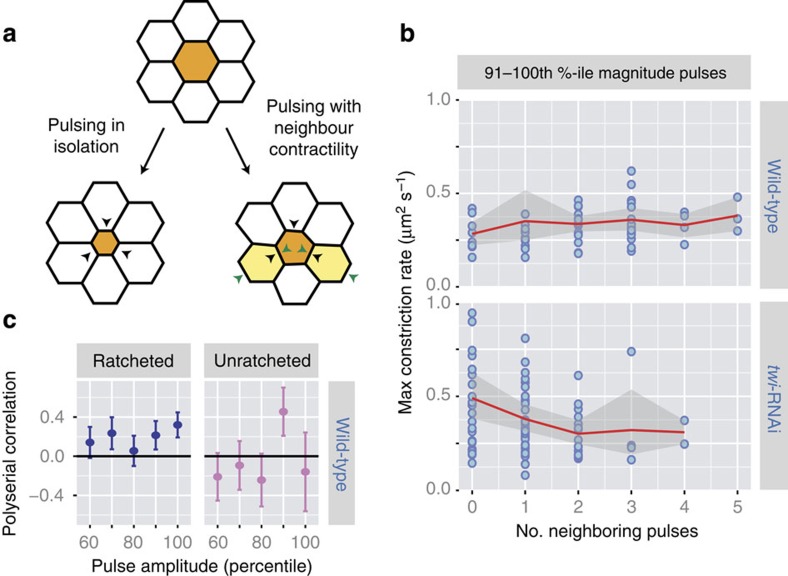
Ratcheting prevents mechanical competition between neighbouring cells. (**a**) Schematics of mechanical competition between contraction pulse in a central cell (orange, black arrowheads) and contraction pulses in its neighbours (yellow, green arrowheads) leading to a lower magnitude of apical constriction in the central cell. (**b**) *twi*-RNAi pulses, but not wild-type pulses, interact competitively. For pulses of similar magnitudes, the maximum constriction rate attained during a pulse (*y* axis) and number of neighbouring pulses (*x* axis) were quantified. For pulses within the 91–100th percentile amplitude-bin, having more neighbouring pulses correlates with less apical constriction in *twi*-RNAi cells. In wild-type cells, neighbouring pulses are not associated with lower constriction rates in the central pulse. The red lines and shaded regions denote the means and s.d.’s, respectively. (**c**) Ratcheted wild-type pulses are associated with cooperative apical constriction. For pulses with similar amplitudes, polyserial correlation scores were quantified between the maximum constriction rate attained during a pulse and the number of neighbouring pulses^24^. Error bars show estimated s.d.’s^24^.

We next asked how pulses are spatially coordinated and how neighbouring cells undergoing contractile pulsing at the same time affect each other’s ability to constrict. Mechanically, neighbouring cells must compete with each other for area reduction due to simple force balance. *In silico* models of ventral furrow cells show that contractile pulses in cells surrounding a central cell can stall each other’s constrictions, leading to lower magnitude of constriction in the central cell (Fig. 6a)^17^. We examined how the magnitude of apical constriction caused by a pulse is affected by having neighbouring cells also undergo contractile pulses. Because apical constriction magnitude depends on the magnitude of myosin pulses (Fig. 1f,g), we initially performed our analysis on contraction pulses within single amplitude-bins. We measured the maximum rate of area reduction attained by a pulse and the number of pulses neighbouring it, defined as any other pulse occurring ±15 s in the nearest neighbour cells. The time window of 15 s was chosen to restrict our analysis to neighbouring cells that are generating force at the same time. For pulses within a single amplitude-bin, we observed that pulses in *twi*-RNAi cells result in less apical constriction if they have more neighbouring pulses, suggesting force balance and thus competition between neighbouring pulses (Fig. 6b, Supplementary Fig. 8b). In contrast, neighbouring pulses do not slow the apical constriction of a central cell in wild-type embryos, suggesting that simple force balance between pulses is not sufficient to explain the interactions between neighbouring pulses (Fig. 6b; Supplementary Fig. 8a). Because pulses in a single amplitude-bin exhibit similar timing, it is unlikely that this measurement is affected by cells with more neighbouring pulses happening later in time. To test the statistical significance of these trends, we used partial correlation analysis, which quantifies the correlation between two variables while removing the effect of a third confounding variable^23^. We used partial correlation to directly quantify the correlation between the number of neighbouring pulses and the magnitude of apical constriction, while removing the influence of pulse amplitude, which we know to directly affect the constriction rate. First, partial correlation confirmed that by controlling for pulse amplitude-bin, we removed the effects of time from correlations seen in the wild-type embryo (Supplementary Table 1). More importantly, focusing on pulses from the top five amplitude-bins, where signal-to-noise ratio of apical constriction is highest, we observed significantly negative correlation in *twi*-RNAi embryos, but a non-negative correlation in wild-type embryos (Table 1). Next, we tested whether different pulse behaviour classes in wild-type cells also exhibited differential trends in cell–cell interaction. We used polyserial correlation, which quantifies correlations between a continuous and a discrete variable, to measure the correlation between the constriction rate and the number of neighbouring pulses surrounding central pulses of either ratcheted or unratcheted class within the same amplitude-bins (see Methods)^24^. Strikingly, we saw that the ratcheted pulses consistently displayed positive correlation scores (Fig. 6c). Partial correlation analysis confirmed that, while unratcheted pulses did not display significant correlation, ratcheted pulses displayed a significant positive correlation (Table 1), suggesting that not only do ratcheted pulses not compete with neighbouring pulses, they on average constrict better when surrounded by neighbouring contractions. Taken together, these results suggest that, ratcheting of pulses in the wild-type ventral furrow not only prevents neighbouring contractile events from competing with each other but might even confer a degree of cooperativity between pulses.

### Ratcheted pulses are enriched in neighbouring contractions

**Figure 7:**
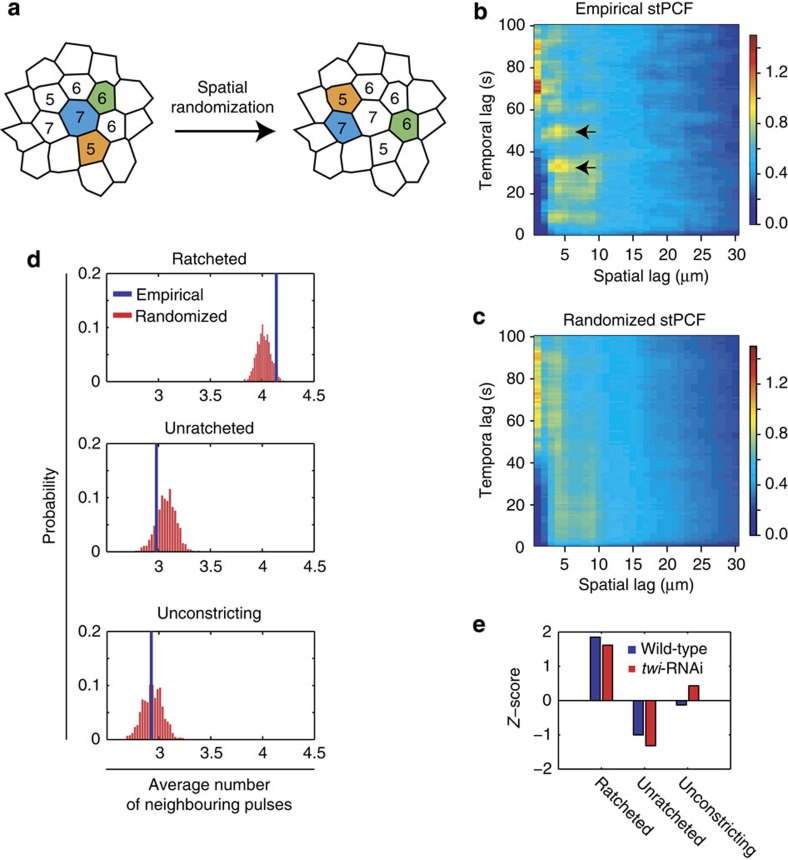
Ratcheting of contractile pulses correlates with enrichment in neighbouring pulses. (**a**) Schematic for the spatial randomization of pulses (coloured cells) while preserving the number of cells locally connected to the pulsing cell (inset number). This randomization was used to calculate whether the frequency of neighbouring pulses was expected by random chance. (**b**,**c**) stPCFs of empirical (**b**) and randomized (**c**) pulsing patterns show an enrichment in neighbouring cells pulsing within 60 s of each other (arrows). The randomized stPCF shows the mean value from 1,000 simulated iterations. (**d**,**e**) Ratcheted pulses have an enrichment in neighbouring pulses in both wild-type and *twi*-RNAi embryos. (**d**) The distributions of the average number of neighbouring pulses from spatial randomization simulations (red) and the empirical frequency (blue) with respect to wild-type pulses of different behaviour classes. (**e**) *Z*-scores of the empirical average frequencies estimated with respect to the randomized distributions for wild-type (blue) and *twi*-RNAi (red) embryos show similar behaviour-dependent enrichment in neighbouring pulses.

Finally, as we saw that the amount of apical constriction attained by a pulse was aided by having neighbouring pulses, we asked whether a central cell’s pulsing could activate or inhibit contractility in its neighbours. We first measured the pattern of pulsing in the embryo by calculating the spatiotemporal pair correlation function (stPCF), which estimates the average probability, given any myosin pulse, that another pulse will occur with *η* microns and *τ* seconds offset (see Methods)^25^. The stPCF showed that nearest neighbour cells tend to have pulses co-occurring within 30–60 s of each other (Fig. 7b,c, arrows). To determine the statistical significance of this effect and whether it depends on the behaviour class of a pulse, we measured the mean frequency of pulse co-occurrence among nearest neighbour cells for central pulses with specific area behaviours. We then generated a null distribution of the mean pulse-neighbour frequency by randomizing the spatial pattern of pulsing within the embryo, while preserving the timing and periodicity of pulses, as well as the local cell connectivity of the pulsing cell (Fig. 7a, Supplementary Fig. 9a–d; see Methods). We saw that the unconstricting pulses have similar number of neighbours in both empirical and random data sets (Fig. 7d). Meanwhile, ratcheted pulses in wild-type tissues displayed enrichment in neighbouring pulses that was statistically significant (*P*<0.03; *Z*-test), and unratcheted pulses on average displayed depletion in neighbouring pulses (Fig. 7d). Strikingly, *twi*-RNAi tissues displayed similar patterns of neighbour enrichment and depletion with respect to pulse class (Fig. 7e, Supplementary Fig. 9e). The difference between the observed pattern of enrichment/depletion surrounding a ratcheted versus an unratcheted cell is significant in both wild-type and *twi*-RNAi tissues, as estimated from the variance of *Z*-scores between repeated simulation measurements (*P*<10^−11^, paired *t*-test; see Methods). These results are consistent with a ‘neighbour-activating’ model of collective cell shape change, where a central cell’s ratcheted contraction pulse could promote contractile events in neighbours, either through mechanical tension or cell shape strain^9^,^11^,^26^,^27^. However, alternative models could also explain this correlation between ratcheting and enrichment in neighbouring pulses (see Discussion). Given the increase in pulse frequency over developmental time, it is possible that transition to ratcheted pulses promotes a higher frequency of pulses. Nevertheless, ratcheted pulses being enriched in neighbouring contractile events suggests that the engagement of a ratchet mechanism promotes locally coordinated contractions in ventral furrow cells. Our data support a model in which intercellular interactions influence dynamic cell behaviours that collectively change tissue shape.

## Discussion

Pulsed or oscillatory actomyosin contractions have been found to underlie many tissue morphogenesis events, ranging from tissue folding^7^, tissue closure^28^,^29^, cell intercalation^30^,^31^,^32^,^33^,^34^, to tissue elongation^35^. How these discrete force-generating events are coordinated in time or across multiple mechanically connected cells to sculpt the overall tissue-level shape has remained poorly understood, especially in the context of tissue folding. Using a computational approach to identify and quantitatively classify contraction events, we exploited the heterogeneity and variation in contraction events and found that during *Drosophila* ventral furrow formation, pulsed contractions exhibit temporal as well as spatial coordination.

**Figure 8:**
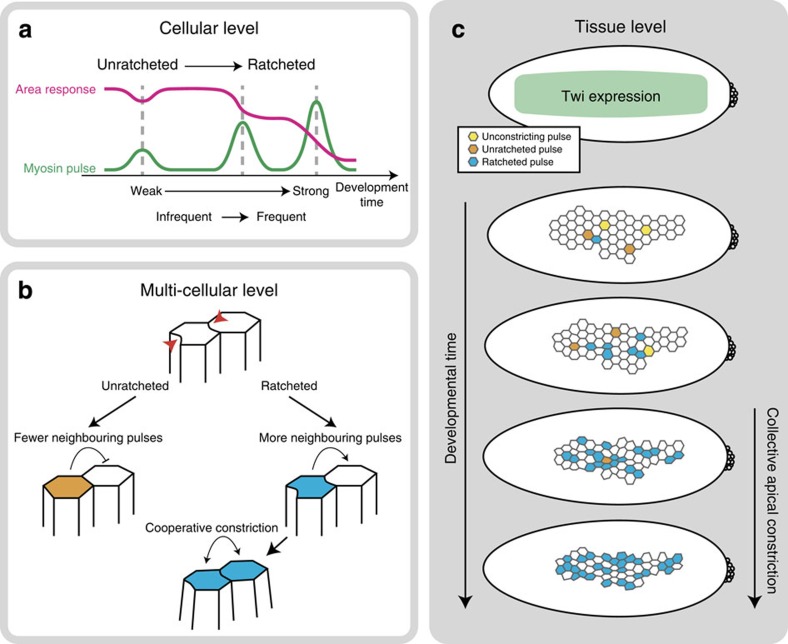
Model for collective apical constriction. (**a**) Individual cell behaviour during ventral furrow formation. Cells transition from weak, infrequent and unratcheted pulses into strong, frequent and ratcheted pulses. (**b**) Local intercellular interactions between ventral furrow cells. Unratcheted pulses have fewer neighbouring pulses, while ratcheted pulses are enriched in neighbouring contractions. Ratcheted pulses cooperatively interact with neighbouring pulses to effect apical constriction. (**c**) Tissue-level behaviour during ventral furrow formation. Twi expression drives transition into collective apical constriction by biasing individual cells to transition from the unconstricting (yellow) and unratcheted (orange) states into the ratcheted (blue) state.

Pioneering studies of cell shape changes during gastrulation suggested that a subpopulation (∼40%) of cells stochastically initiate constriction, followed by the constriction of the remaining unconstricted cells^5^,^8^. It has been proposed that a mechanical signal could trigger this transition from stochastic to collective constriction^9^. Here we found that, while there is no evidence of subpopulations of cells initiating constriction at different times, Twi expression in the ventral furrow drives individual cells to undergo a sequence of contractile behaviours that eventually results in collective tissue contraction and folding of the ventral furrow (Fig. 8a,c). First, we showed that cells in wild-type embryos undergo an ordered transition from low-amplitude to higher-amplitude myosin pulses during invagination. Second, we found that unconstricting, unratcheted and ratcheted pulses were also ordered, with unconstricting and unratcheted pulses occurring first and followed by ratcheted pulses. Third, we measured the transition frequencies in individual cells to show that biased transitions occur at the cell level. In Twi-depleted embryos, pulses of different magnitude and different classes occurred with overlapping distributions and failed to exhibit a clear order. At the cell level, Twi depletion increased the frequency of inappropriate reverse transitions in pulse behaviour. Twi likely mediates the transition in the contractile state through the Rho pathway, by promoting increasing apical localization of Rok and its organization into a stable signalling complex at the medioapical centre of the cell apex, which could be guiding the formation of persistent myosin structures that mechanically couple multiple cells in the tissue. In addition, Twi could be important for the proper function of adherens junctions, which could be important to maintain the coupling of myosin structures to the junction^15^,^16^,^36^. In conclusion, Twi depletion does not result in a wholly novel unratcheted behaviour, but instead disrupts the ability for ventral furrow cells to transition from the unratcheted to the ratcheted state and to stay in the ratcheted state, which ultimately disrupts tissue folding. This transition in the ratcheting behaviour resembles that seen in apically constricting amnioserosa cells during dorsal closure^28^,^29^,^37^,^38^. Whereas in dorsal closure, this transition occurs on the order of 1 h, in the ventral furrow, this transition happens within minutes. Therefore, our study suggests that the unratcheted-to-ratcheted transition is a general mechanism that coordinates multicellular dynamic force generation over a range of timescales.

We also demonstrated that cells undergoing ratcheted contractile pulses do not compete with each other, suggesting that the engagement of the ratchet is important for neighbouring cells to coordinately constrict. *In silico* models based on mechanical interactions between cells predicted that neighbouring cells contracting at the same time should pull on each other through force balance, thereby reducing their amount of cell shape constriction attained during their respective contraction pulses^17^. However, our quantitative analysis showed that, while the signature of competition between neighbouring pulses is present in Twi-depleted tissues, wild-type tissues do not exhibit competition. Furthermore, when we focus our analysis on ratcheted wild-type pulses, we see a significant positive correlation between the number of neighbouring pulses and the constriction rate, suggesting cooperation instead of competition (Fig. 8b). One model for how this occurs is that ratcheted pulses leave the cell with cytoskeletal structures spanning the apical domain (persistent myosin), which bear tension. These structures could mechanically couple neighbouring constricting cell cortices, allowing external forces to be propagated across the cell cluster instead of locally slowing constriction within the cluster. Consistent with this model, Twi depletion, which disrupts the predominance of ratcheted pulses, lowers epithelial tension in the ventral furrow^16^.

Lastly, we demonstrated that ratcheted pulses have a higher probability of having neighbouring contractile pulses than expected by chance. Interestingly, this effect is also observed in Twi-depleted embryos. We previously speculated that neighbouring contractile pulses could destabilize each other, leading to unratcheted constrictions^7^. In contrast, our current study suggests that unratcheted pulses are in fact depleted of neighbouring contractions, while ratcheted pulses are enriched in neighbouring contractions. One model consistent with these results is that pulsing cells are activating contractions in their immediate neighbours through a mechanosensitive process^9^,^11^,^26^,^27^ (Fig. 8b). In this model, ratcheting could be crucial for sustaining local tension in the vicinity of the cell in order to strengthen the mechanical signal or its propagation. Alternatively, locally clustered pulses could be more resistant against being stretched out by global tissue tension, yielding the observed pattern of enrichment around ratcheted pulses. In this case, neighbouring pulses could act as a temporary ‘corset’ that serves to ratchet a cell in a non-cell-autonomous manner. We speculate that this secondary ratchet mechanism could contribute to ratcheted pulses in *twi*-RNAi cells, where there are ratcheted pulses despite having low average myosin persistence (Fig. 1l, Supplementary Fig. 5g). Although the molecular or mechanical basis for enrichment of neighbouring pulses is not known, our results demonstrate that intercellular interactions that promote contractility occur during constriction and depend on pulses being ratcheted. Overall, the transition in individual cells to having ratcheted pulses could increase the frequency of neighbouring pulses and prevent competition between pulses. Thus, Twi-dependent changes in the properties of contractile events in all cells, not the number of cells having initiated constriction, would promote the transition to collective cell constriction.

## Methods

### Fly stocks

The following fluorescent constructs are used in this paper: *sqh*-Gap43::mCherry (membrane marker driven by myosin regulatory light chain promoter)^16^, *sqh*-Myosin::GFP (Sqh::GFP)^39^ and *ubi*-GFP::Rok (gifts from Y. Bellaïche, Institut Curie, Paris, France)^40^. Gap43::mCherry/CyO; Myosin::GFP flies were crossed to sqh^AX3^; Myosin::GFP flies and non-CyO females were collected. Embryos from Gap43::mCherry/+; Myosin::GFP/Myosin::GFP females crossed with OregonR males were imaged. Ubi-GFP::Rok/X flies were crossed to Gap43::mCherry/CyO and non-CyO females were collected. Embryos from Ubi-GFP::Rok; Gap43::mCherry females crossed to sibling or OregonR males were imaged to generate Rok and membrane-labelled embryos.

### Live imaging

Embryos were dechorionated with 50% commercial bleach and washed with water. They were mounted ventral side up on a glue-coated slide. No. 1.5 coverslips were glued to either side of the embryo to avoid its compression. A No. 1 coverslip was added on top to create a chamber, into which halocarbon 27 oil was added for imaging. Glue was generated by dissolving double-sided tape in hexane.

Images were acquired on a Zeiss LSM 710 confocal microscope with a × 40/1.2 Apochromat water objective (Carl Zeiss). Two-colour images were acquired simultaneously. A pinhole of two Airy units was used. For GFP, a 488-nm Argon ion laser was used for excitation and a 493- to 557-nm bandpass filter used for emission. For mCherry, a 561-nm diode laser was used for excitation and a 572- to 700-bandpass filter used for emission. To generate sufficient statistical power, five embryos were imaged per experimental condition.

### dsRNA injection

dsRNA was generated using the Invitrogen MEGAscript T7 transcription kit and purified using phenol–chloroform RNA extraction and resuspended in 0.1 × PBS^7^. For injection, embryos were dechorionated in bleach and desiccated for 4–6 min in a chamber with anhydrous Drierite. They were mounted ventral-side up on a glass slide and covered in injection oil, a 3:1 mixture of halocarbon 800 and halocarbon 27 oils. Borosilicate glass capillary needles were used for injection. To ensure sufficient knockdown of the target gene, all dsRNA injections were given in the blastoderm stage, 2.5–3.5 h before gastrulation. After injection, the injection oil was removed and replaced with halocarbon 27 oil. The embryos were stored in the dark at room temperature until imaging. Control embryos were injected with × 0.1 PBS.

### Primers used

The following primers were used to generate *twi* dsRNA: F: 5′-TAATACGACTCACTATAGGGGCCAAGCAAGATCACCAAAT-3′; R: 5′-TAATACGACTCACTATAGGGGACCTCGTTGCTGGGTATGT-3′.

### Image processing and segmentation

Image stacks were pre-processed using low-pass Gaussian filters (kernel size=1px). They were then imported into Embryo Development Geometry Explorer (EDGE) for segmentation of cell boundaries^41^. Owing to the curvature of the embryo, a z-slice 3–4 μm below the most apical slice was used for segmentation. For injected embryos, a z-slice of 6–7 μm below the apex was used because *twi*-RNAi cells have disordered shapes at their very apex. This difference is unlikely to change our results because we have analysed control-injected embryos at 6–7 μm below the apex and obtained identical results to our analysis using a 3- to 4-μm segmentation depth. For all data sets, no data were included in the study if the tissue has invaginated beyond 3 μm of its original apex position.

To subtract the nonapical, cytoplasmic signal in the myosin channel, a cytoplasmic section of the myosin images was chosen from a more basal z-slice of the image, 8–9 μm from the apex. A mean and a s.d. was estimated from this basal section, and a threshold was empirically determined to be 2 s.d.’s above the mean intensity. This threshold was then used to subtract background signal from the entire myosin image stack. As the surface of the embryo is curved, a maximum intensity Z-projection was used on the thresholded myosin stacks, up to the segmented membrane z-slice. This final processed image was then imported into EDGE, where the myosin intensity within a single cell was measured as the total intensity of the processed myosin image contained within the segmented boundaries of that cell. Using this framework, we also measured other properties of individual cells as a function of time, including the apical area, the centroid of the cell and next-neighbours of the cell within the epithelium.

### Temporal alignment of embryos

The onset of average apical area decrease (Supplementary Fig. 6a, dotted line) was manually determined and used to align the developmental progression of wild-type embryos. *twi*-RNAi embryos, which lack a clear transition between constricting and nonconstricting states, were temporally aligned by the point at which the average apical area is equal to 40 μm^2^ (Supplementary Fig. 6b, dotted line), the average apical area for cells at the end of cellularization in both wild-type and *twi*-RNAi embryos.

### Pulse detection and curation

An iterative multiple-Gaussian fitting algorithm was designed to detect temporal peaks in the myosin intensity signal from each cell. MATLAB’s nonlinear least-squares optimization implementation *lsqcurvefit* was used to iteratively fit an increasing number of Gaussians plus an exponential background, to the myosin signal. The F-test for variance equality (*α*=0.01) was used as the stopping criterion, such that if the ratio of reduced residuals of the *n+1*-Gaussian-plus-background model to the *n*-Gaussian-plus-background model fails the F-test, the algorithm exits and reports the *n*-Gaussian-plus-background model as the best-fit model. Finally, the best-fit *n*-Gaussian-plus-background model was compared with an exponential background-only model. To model the typical duration of a pulse^7^, the *σ* of the Gaussian models was restricted to between 10 and 30 s during least-squares optimization.

To verify these putative pulses, we used the MTrackJ ImageJ/FIJI plug-in to manually track when pulses occur within each cell in the embryo. These manual tracks were then mapped on the putative tracks when they overlapped for more than two frames. We then determined the error rate of peak-fitting by categorizing the mapping between the fitted pulses and the tracked pulses; the five categories were as follows: (1) one to one, (2) missed by fitting, (3) added by fitting, (4) split by fitting and (5) merged by fitting (Supplementary Fig. 2a). Of the categories, only one to one corresponds to a correct identification, while the four other categories are different ways the algorithm yields misidentifications.

A user examined the image sequences and time series quantifications of each misidentified event to manually determine whether a misidentified Gaussian fit was to be kept or not. For pulses that were missed by the Gaussian fitting, parameters were estimated by hand.

### Pulse amplitude binning

Pulse amplitudes or magnitudes were determined on the basis of Gaussian amplitude fitted for each particular pulse, and therefore is a reflection of the maximum myosin intensity reached during a pulse, having subtracted the background signal. Because the myosin intensity values are not directly comparable across separate embryos and movies, we determined the intraembryo percentile ranking of each pulse’s amplitude. We then collated pulses with similarly ranked amplitudes across embryos and grouped them into 10 percentile (or %-ile) bins.

### Pulse-centric measurements

To examine dynamic data (myosin intensity, apical area, and so on) at a single-pulse level, we took subsequences of the time series of interest centred around the Gaussian centres of individual pulses. Time series with different image-acquisition rates were interpolated using linear interpolation to a standard frame rate, set at the average frame rate of all movies to be examined. Linear interpolation was also used to impute missing values within a time series, although no extrapolation was performed(that is, beyond the beginning or the end of the movie). The subsequence was then truncated to be 30 s before and 45 s after the Gaussian centre of the pulse. This timeframe was picked to minimize overlap between consecutive pulses (<6% of pulse timeframe overlap) as well as maximizing the amount of information about area-response behaviour that occur after the maximum of the pulse. In the case of apical area, to calculate the local change in apical area, we subtracted the mean apical area within the pulse timeframe to obtain the mean-centred, relative change in apical area, which we termed the *area response*.

### Pulse behaviour classification

To detect whether each pulse induced a ratcheted constriction, we used FCM clustering to classify the area response of a pulse. FCM is an unsupervised clustering method similar to the *k*-means algorithm. Instead of a hard membership assignment of a datum to a cluster, the FCM algorithm assigns a fuzzy membership on the basis of the distance between the datum to each centroid; similarly, the centroid update is a weighted average of fuzzy memberships of all data^18^. To classify the shape of the area response and not necessarily its magnitude, we pre-processed the data by normalizing the area-response curves by their s.d., and used the Euclidean distance as the distance metric. Data with missing values were disregarded by the algorithm. Finally, a datum was assigned to the centroid to which it had the maximum membership after convergence. The MATLAB implementation *fcm* from the Fuzzy Logic Toolbox was used with random initial seeds.

To find the appropriate number of cluster centres used to seed the FCM algorithm, we used PCA, which has been shown to be closely related to the *k*-means clustering algorithms^42^. PCA shows that the first three principal components explain >92% of the total variance (Supplementary Fig. 4c). We varied the number of seed clusters and quantified the average distortion, or the distance between cluster members to their respective cluster centroid, as well as the average silhouette value, which quantifies the normalized difference of the distance between each datum to its assigned cluster centroid and the nearest neighbouring nonmember cluster centroid. For both analyses, better clustering of data will yield lower values, with ‘natural’ clusters in the data exhibiting a ‘kink’ or ‘elbow’ at that cluster number^43^,^44^. For our data, both distortion and silhouette analyses show a smooth asymptotic decrease, with no strong ‘kink’ at any particular cluster number, indicating that the data are not naturally clustered but most likely exhibits a continuous spectrum of behaviour (Supplementary Fig. 4d). In addition, graphically representing the data on a PCA plot reveals no strong visual clustering (Supplementary Fig. 4b). Therefore, the cluster number chosen in this study (*k*=3) can be thought of as separating the extreme parts of a continuum, instead of indicating that there are three disparate area-response behaviours. Owing to the nondeterministic nature of the FCM algorithm, we repeated the clustering procedure 1,000 times with random initial seeds. We found that for *k*=3 the clustering was very stable, as quantified by the Rand index (*RI*=1 between all FCM replications)^45^.

### Myosin persistence

The persistence of myosin after a pulse was defined as the difference in the minimum myosin intensity before the pulse centre and the minimum myosin intensity after the pulse centre, normalized by the mean myosin intensity throughout the pulse (Fig. 2b).

### Statistical analysis and testing

All statistical tests were performedin MATLAB (The MathWorks) or R (The R Project for Statistical Computing).

Two-sided, two-sample Kolmogorov–Smirnov tests were used to compare empirical distributions. Correlation coefficients reported were Pearson’s correlations, unless noted otherwise. The *P* values reported were calculated against a null-hypothesis of no correlation, calculated using Fisher’s *Z*-transform.

Hartigan’s *dip* test was used to test whether distributions exhibited more than a single mode (null hypothesis is unimodality)^21^.

Polyserial correlation from the *polycor* package in R was used to quantify the correlation between a continuous (*X*) and a ranked variable (*Y*), as in the case of apical constriction magnitude and the number of neighbouring pulses^24^,^46^. Briefly, the polyserial correlation (*ρ*) reported the maximum-likelihood estimate of the Pearson correlation between *X* and *Y*, accounting for the fact that *Y* is indirectly observed as a ranked variable. The s.d. of the maximum-likelihood estimate of *ρ* is given by the two-step method of numerical variance estimation^24^.

Partial correlation was calculated using the *ppcor* package in R. Unless noted otherwise, *P* values were calculated against a null hypothesis of no correlation^23^. Briefly, partial correlation correlates two variables *X,Y*, while removing the influence of a confounding variable, *Z*. It is defined as the correlation of the residuals *R*_*XZ*_ and *R*_*YZ*_ resulting from the linear regression between *X* and *Z*, and *Y* and *Z*, respectively.

### Jensen–Shannon divergence

JSD is a measurement of the dissimilarity of two probability distributions on the basis of the Kullback–Leibler divergence. For two probability distributions, *R*(*x*) and *Q*(*x*), the JSD is defined as:




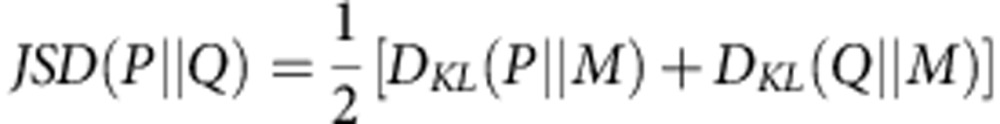




where 
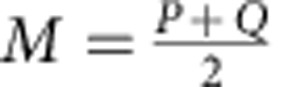
, and *D*_*KL*_(*P*||*Q*) is the Kullback–Leibler divergence, defined as:




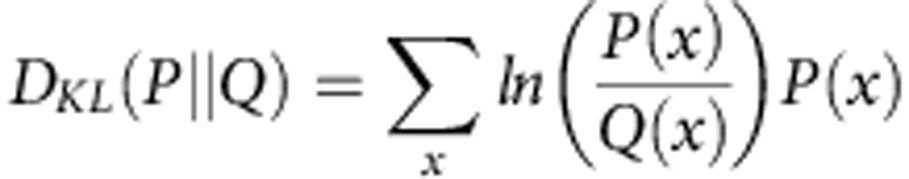




The JSDs of pulse timing between pulses of different magnitudes were measured using distributions estimated from histograms of pulse timings (Fig. 4d,g).

### Spatiotemporal pair correlation function

The sequence of pulses within an individual embryo can be modelled as a sequence of spatiotemporal points, with the *i*-th pulse being summarized by its spatial coordinates ***s***_***i***_=(*x*_*i*_, *y*_*i*_), corresponding to the centroid of the pulsing cell, and its temporal coordinate *t*_*i*_ corresponding to its Gaussian centre.

The stPCF of a spatiotemporal point process with *N* observed points, {(***s***_***i***_, *t*_*i*_)}, *i*=1,…,*N*, is defined as^25^,^47^:




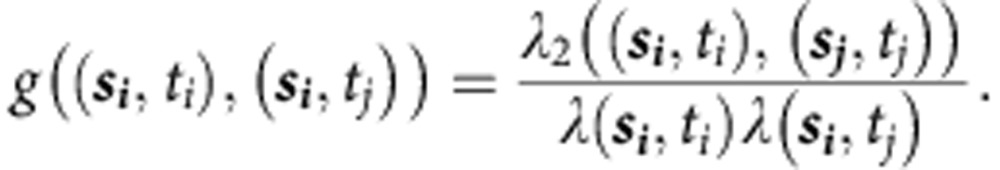




*λ*(***s***,*t*) describes the first-order intensity of the point process, or informally, the spatiotemporal density:




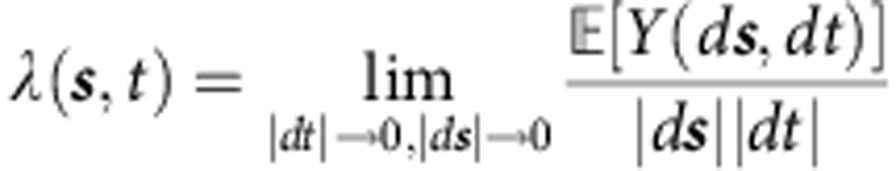




where *Y*(d***s***,d*t*) is the number of events occurring within a spatial region d***s*** and time interval d*t*.

Similarly, *λ*_2_((***s***_***i***_,*t*_*i*_),(***s***_***j***_,*t*_*j*_)) describes the second-order intensity of the process:




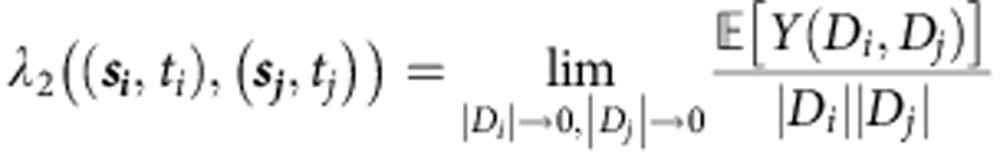




where *D*_*i*_=d***s***_***i***_ × d*t*_*i*_ describes a ‘voxel’ of time–space centred around the point (***s***_***i***_,*t*_*i*_), and *Y*(*D*_*i*_,*D*_*j*_) is the number of times two events co-occur with spatial separation |***s***_***i***_–***s***_***j***_| and temporal separation |*t*_*i*_–*t*_*j*_|.

Estimation of the stPCF was made using the R language package *stpp*, which uses nonparametric kernel density estimators^47^. For our analysis, we used a Gaussian temporal kernel and a box spatial kernel; all kernel sizes were chosen to minimize the mean square error.

### Pulse spatial randomization

Spatially random pulsing data sets were simulated by randomizing the spatial location of individual pulses within an embryo. For each pulse found within an embryo, the local cell connectivity (that is, number of cells neighbouring the pulsing cell at the Gaussian centre of the pulse) is determined, and we draw up a list of cells with the same local cell connectivity. We then choose a random candidate cell from this list. To preserve the frequency between consecutive pulses, we first estimate the distribution of empirical pulse frequencies using a gamma distribution, which we use to determine the probability of accepting placing the pulse in the candidate cell based on the time interval to the previous pulse, in a strategy similar to rejection sampling. This simulation strategy produces an overall distribution of pulse frequencies indistinguishable from the empirical distribution (*P*>0.25, KS test, Supplementary Fig. 9a), as well as preserving the developmental increase in pulse frequency (Supplementary Fig. 9b,c). By design, the local cell connectivity is also preserved (Supplementary Fig. 9d). To reduce edge effects, cells at the boundary of the segmented tissue were excluded from the list of ‘centre’ cells from which the average number of neighbouring pulses were estimated, but could still be counted as a ‘neighbour’ to another, more interior, ‘centre’ cell.

Randomizations were repeated for 1,000 iterations to obtain a null distribution of average neighbour-pulse frequency for the three behaviour classes of pulses. *Z*-scores of the empirical mean frequencies were calculated with respect to normal parameters estimated from this null distribution. *Z*-scores were used in place of *t*-scores due to the large resampling size.

### Comparison of *Z*-scores between pulse behaviour classes

The *Z*-scores from different pulse behaviour classes were compared by subsampling from the 1,000 iterations to estimate a variance of *Z*-scores. Paired *t*-test was used to test for difference in the mean between the *Z*-scores found for ratcheted pulses and unratcheted pulses between each subsample.

### Code availability

All codes used in the study will be made available at request. A Git repository is available at: https://github.com/xies/pulse_finding.

## Additional information

**How to cite this article:** Xie, S. & Martin, A.C. Intracellular signalling and intercellular coupling coordinate heterogeneous contractile events to facilitate tissue folding. *Nat. Commun.* 6:7161 doi: 10.1038/ncomms8161 (2015).

## Supplementary Material

Supplementary Information Supplementary Figures 1-9 and Supplementary Table 1

Supplementary Movie 1 Pulsed contractions during wild-type ventral furrow formation

Supplementary Movie 2 Pulsed contractions during twi-RNAi ventral furrow formation

## Figures and Tables

**Table 1:** Ratcheted pulses do not compete with neighbouring pulses.

**Pulses**	**Partial correlation (*****P*** **value)**
Wild-type pulses of all classes	*ρ*=0.103 (*P*<0.1)
*twi*-RNAi pulses of all classes	*ρ*=−0.200 (*P*<10^−4^)
Wild-type unratcheted pulses	*ρ*=−0.0598 (*P*>0.6)
Wild-type ratcheted pulses	*ρ*=0.210 (*P*<0.005)

Partial correlation scores between the number of neighbouring pulses and maximum apical constriction attained, controlling for the effect of pulse amplitude. Data shown for 51–100th percentile pulses. *P* value is against a null hypothesis of no correlation.
