# Supplementary material for: Intracellular signalling and intercellular coupling coordinate heterogeneous contractile events to facilitate tissue folding
Source: Nat Commun. 2015 May 26;6:7161. doi: 10.1038/ncomms8161 (PMC4445457; doi:10.1038/ncomms8161)
Supplement: Supplementary Information — Supplementary Figures 1-9 and Supplementary Table 1 [file ncomms8161-s1.pdf]

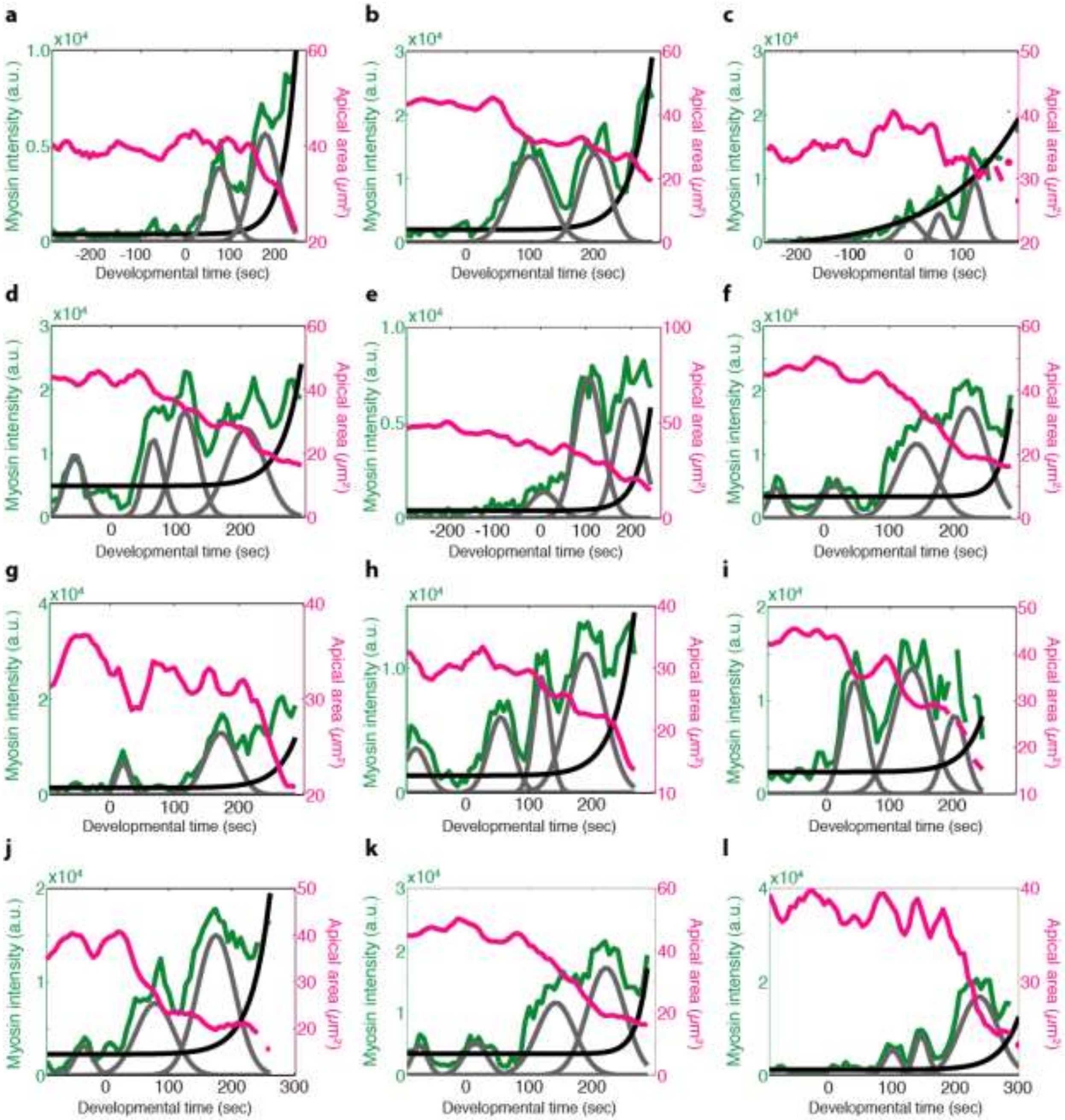

Supplementary Figure 1. **Examples of pulse fitting results.**

a-l. Pulses identified from representative cells. Myosin intensity (green) and apical area (magenta) were quantified from segmented cells. Gaussian peaks (grey) and an exponential background (black) were fitted to the myosin time-series.

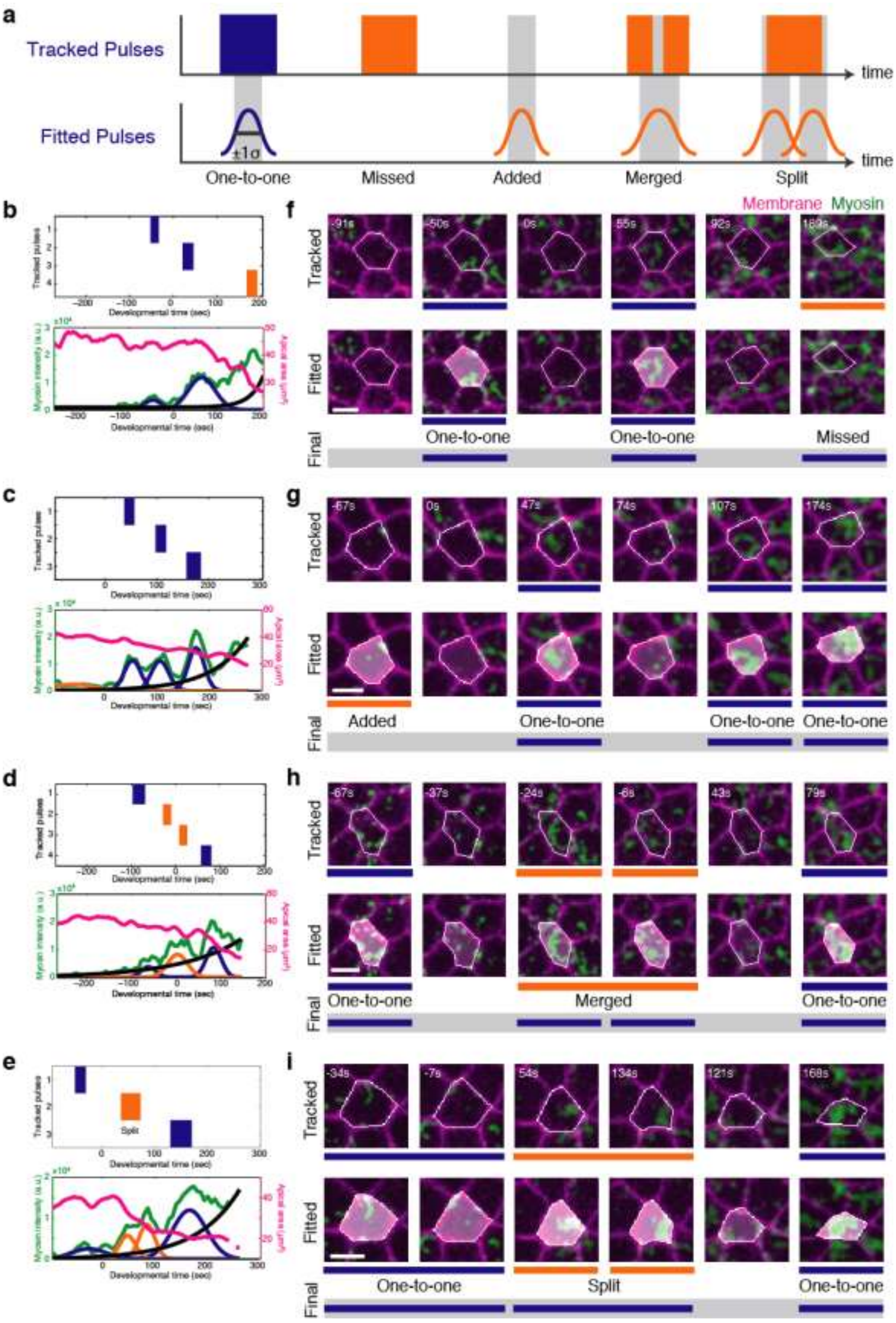

a
Tracked Pulses
Fitted Pulses
time
time
±1σ
One-to-one
Missed
Added
Merged
Split
b
c
d
e
Split
f
g
h
i
Membrane Myosin
Tracked
Fitted
Final
-91s
-50s
0s
55s
92s
One-to-one
One-to-one
Missed
-67s
0s
47s
74s
107s
174s
Added
One-to-one
One-to-one
One-to-one
-67s
-37s
-24s
-6s
43s
79s
One-to-one
Merged
One-to-one
-34s
-7s
54s
134s
121s
168s
One-to-one
Split
One-to-one

Supplementary Figure 2. **Manual curation of computationally fitted myosin pulses.**

a. Schematic of matching computationally fitted pulses to manually tracked pulses (see **Methods**). Tracked pulses were manually identified from movies of ventral furrow formation (*top*); fitted pulses were automatically detected as peaks in the myosin intensity time-series (*bottom*). Fitted and tracked pulses were then matched to each other by looking for overlaps in when they occur, using $\pm 1\sigma$ from the Gaussian mean as the time-frame of a fitted pulse. One-to-one matches (blue) denote agreements between fitted and tracked pulses. Four categories of erroneous identifications (orange) include: added, missed, merged, and split pulses. The final dataset includes all fitted and tracked pulses and were visually confirmed.

b-e. Representative data for the four different error categories: missed (b), added (c), merged (d), and split (e). Top panel shows the timing of the tracked pulses. Bottom panel shows the myosin intensity (green) and apical area (magenta) time-series, as well as the fitted background (black) and Gaussian peaks. The correctly identified fitted peaks are shown in blue while the erroneous identification(s) is shown in orange.

f-i. Representative images for the four different error categories: missed (f), added (g), merged (h), and split (i), corresponding to the data displayed in (b-e). Top montage shows the segmented cell membrane (magenta) and myosin channels. The tracked pulses and when they occur are shown as bars below the image. Bottom montage shows the fitted pulses as white overlay on the segmented cell, as well as bars below the image. Blue bars correspond to one-to-one matches while orange bars correspond to erroneous identifications. The final pulse datasets are shown in the last row. Scale bars are 5µm.

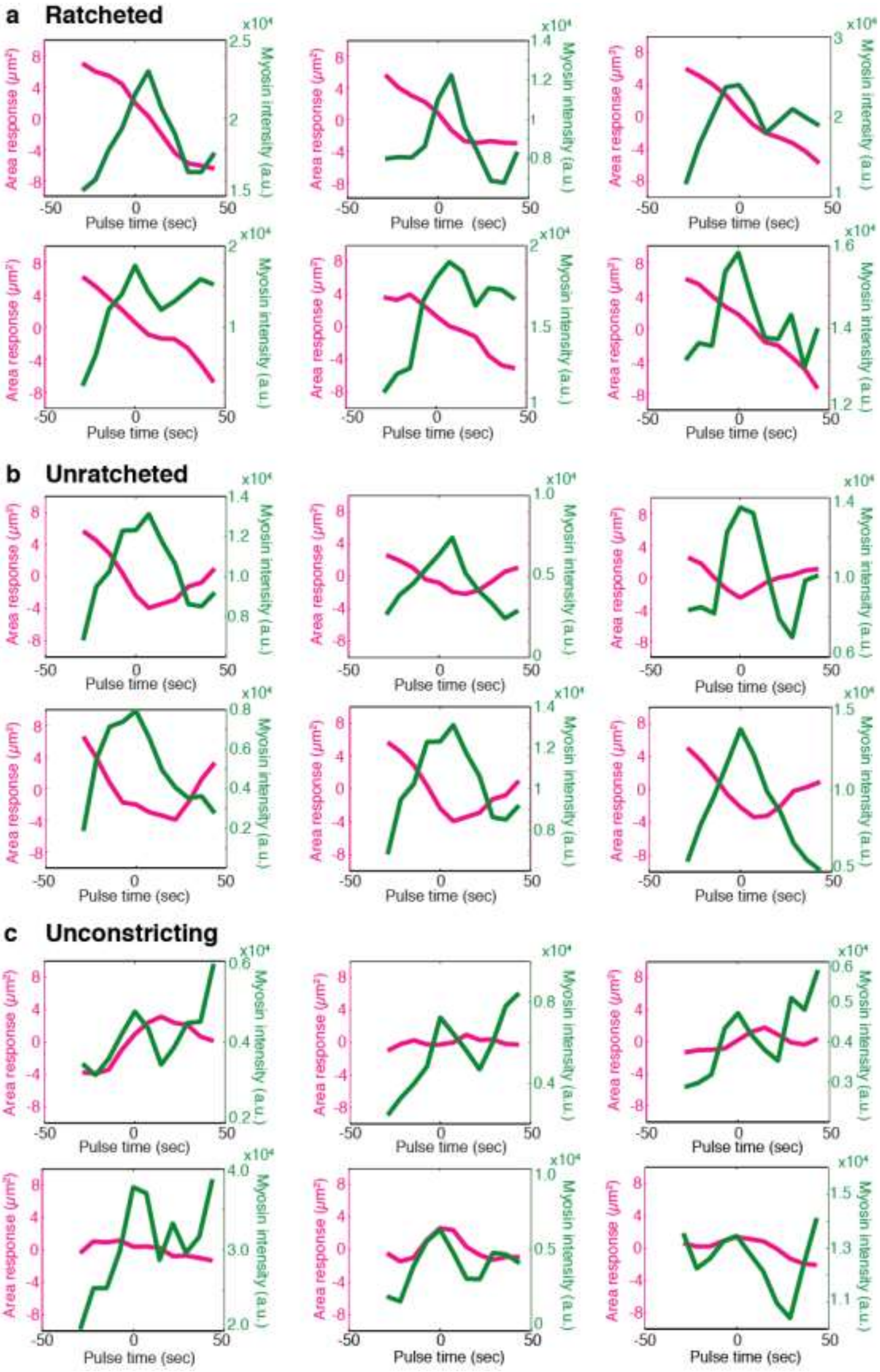
a Ratcheted
b Unratcheted
c Unconstricting
Area response (μm²)
Myosin intensity (a.u.)
Pulse time (sec)
x10⁴

Supplementary Figure 3. **Pulses exhibit heterogeneous behaviours that can be categorized to three classes: ratcheted, unratcheted, and unconstricting.**

a-c. Examples of pulses in the three behaviour classes: (a) ratcheted, (b) unratcheted, and (c) unconstricting.

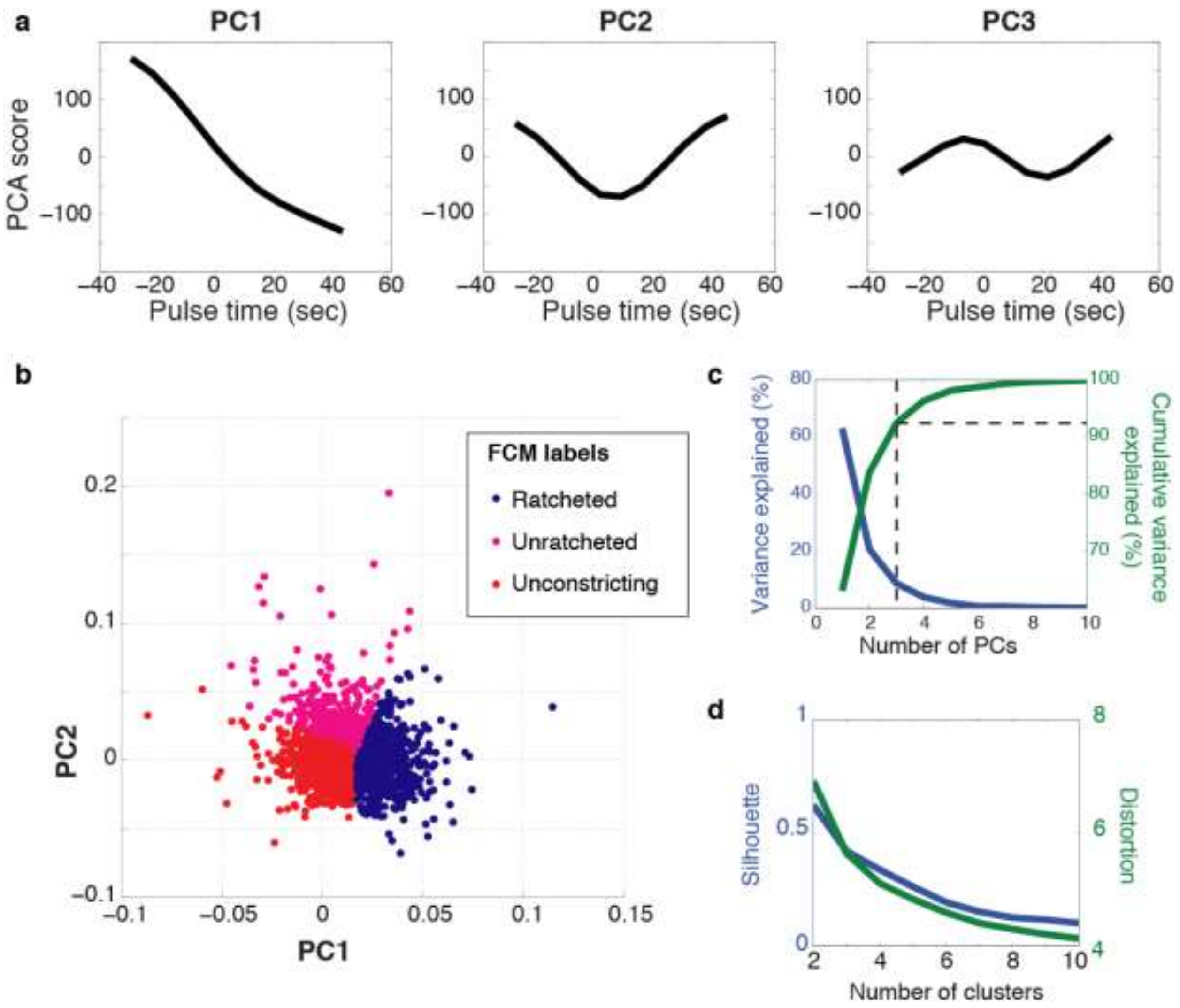

Supplementary Figure 4. **Principal component analysis of area responses to pulses from wild-type and *twi*-RNAi embryos supports the use of three clusters.**

a. The top three principal component scores identified by the covariance PCA of wild-type and *twi*-RNAi pulses are qualitatively similar to the average within-class behaviours identified by fuzzy *c*-means (FCM) (**Figure 1i-k**).

b. FCM-identified behaviour classes partition the dataset along principal components. Distribution of data points in PC space also shows that clustering partitions a continuum of behaviours instead of identifying well-separated groups.

c. The cumulative explained variance of three PCs exceeds 90% of total variance. The percentage of total variance explained by each PC (blue) and the cumulative variance explained (green) are shown. The dotted line marks a cluster number of 3 used in the study.

d. Distortion and silhouette analysis, which assesses the compactness and separability of clusters (see **Methods**) do not display an appreciable "kink" or "elbow" at a cluster number, indicating that in the space of the distance metric used by the study (Euclidean distance) the area response behaviours are a continuum and not well-separated.

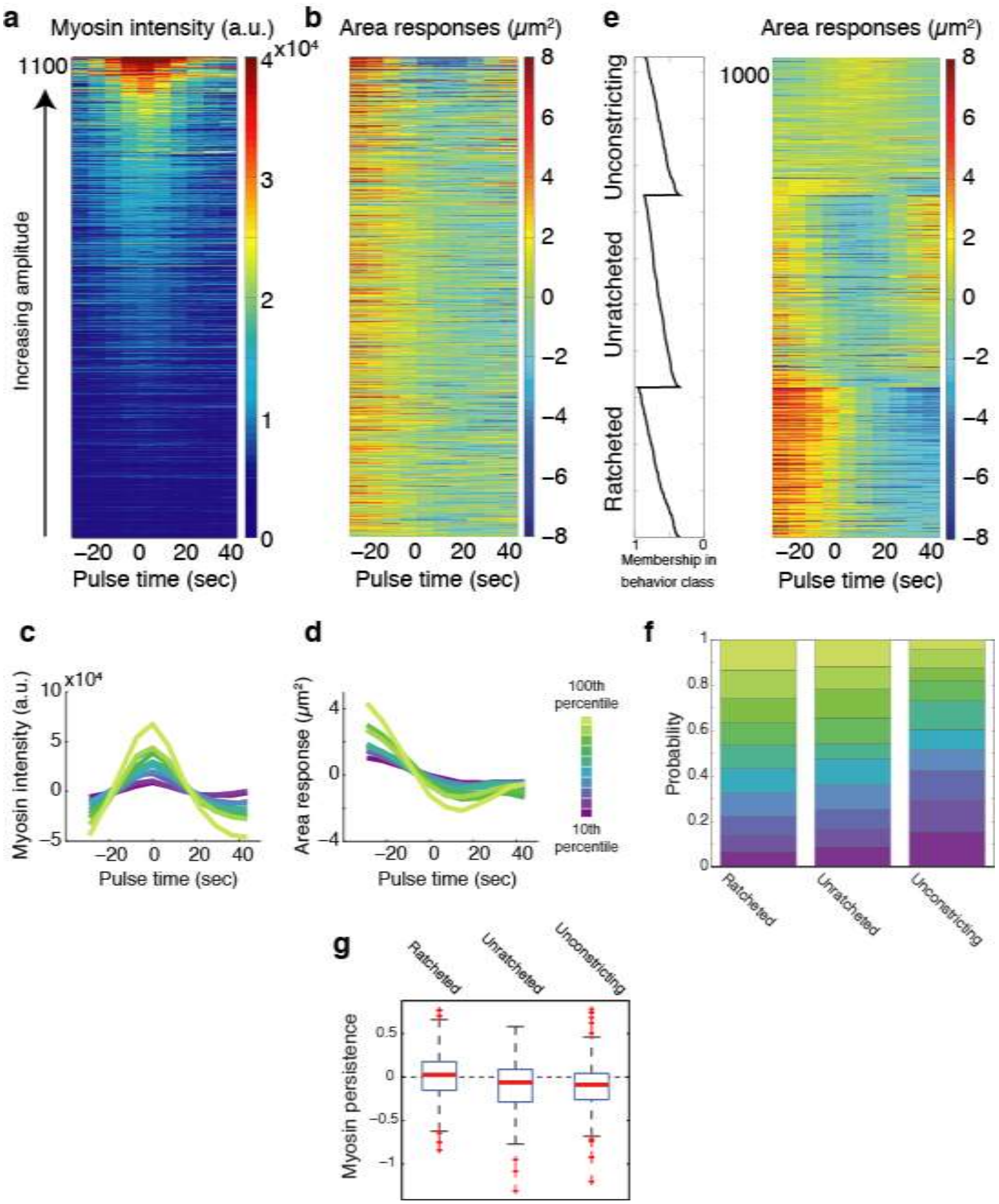

Supplementary Figure 5. **Pulses identified from *twi*-RNAi cells**

a-b. Myosin pulses ($n = 1127$) were identified from *twi*-RNAi embryos ($n = 5$) and sorted by increasing amplitude. Heatmap of the myosin intensity (a) and change in apical area (b) for each pulse.

c-d. Average mean-centred myosin intensity (c) and average mean-centred area response (d) of pulses in different amplitude-bins. Colours denote the percentile-ranking in pulse amplitude (amplitude-bin) ($n \geq 110$ for each colour).

e. Pulses were clustered into three categories according to the apical area behaviour. Heatmap shows the area responses to pulses ($n = 1032$) clustered by FCM into ratcheted, unratcheted, and unconstricting classes (right). Within each class, pulses were also sorted by the degree of membership of each area response within their respective category (left). Pulses with missing data points were not categorized and not shown ($n = 95$).

f. Probability of pulses existing in each amplitude-bin for ratcheted, unratcheted, and unconstricting classes of pulses in *twi*-RNAi embryos.

g. Myosin persistence (see **Figure 2b** and **Methods**) measured for *twi*-RNAi pulses from the ratcheted, unratcheted, and unconstricting classes. Red bars represent sample medians and boxes demarcate the 25th and 75th percentiles.

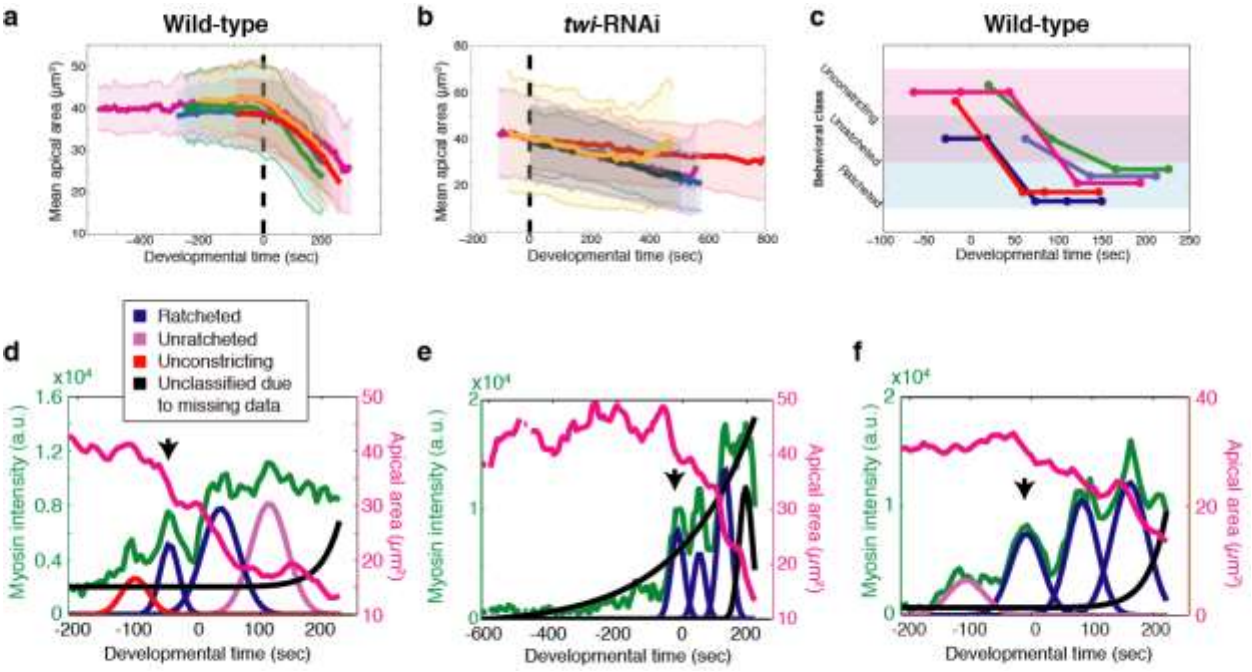

Supplementary Figure 6. **Tissue-level temporal dynamics**

a. Wild-type embryos ($n = 5$) were temporally aligned by the onset of mean apical area reduction. Colours indicate different embryos. Error bars represent standard deviations.

b. *twi*-RNAi embryos ($n = 5$) were temporally aligned by the point at which the average apical area is equal to $40\mu m^2$ (see **Methods**). Colours indicate different embryos. Error bars represent standard deviations.

c. Individual wild-type cells transition from unratcheted and unconstricting pulses to ratcheted pulses. The transitions between pulse behaviour class throughout developmental time are shown for five individual cells (coloured lines).

d-f. Early ratcheted pulses result in lasting cell constrictions. Examples of three different cells where ratcheted pulses occur before the onset of tissue contraction ($t = 0$) are shown (arrows). The apical area (magenta) and apical myosin intensity (green) were quantified. Fitted background in black and fitted pulses are shown in colours according to the legend. The reversed transition from ratcheted to unratcheted pulse shown in (d) is an example of an infrequent event (see **Figure 4l**).

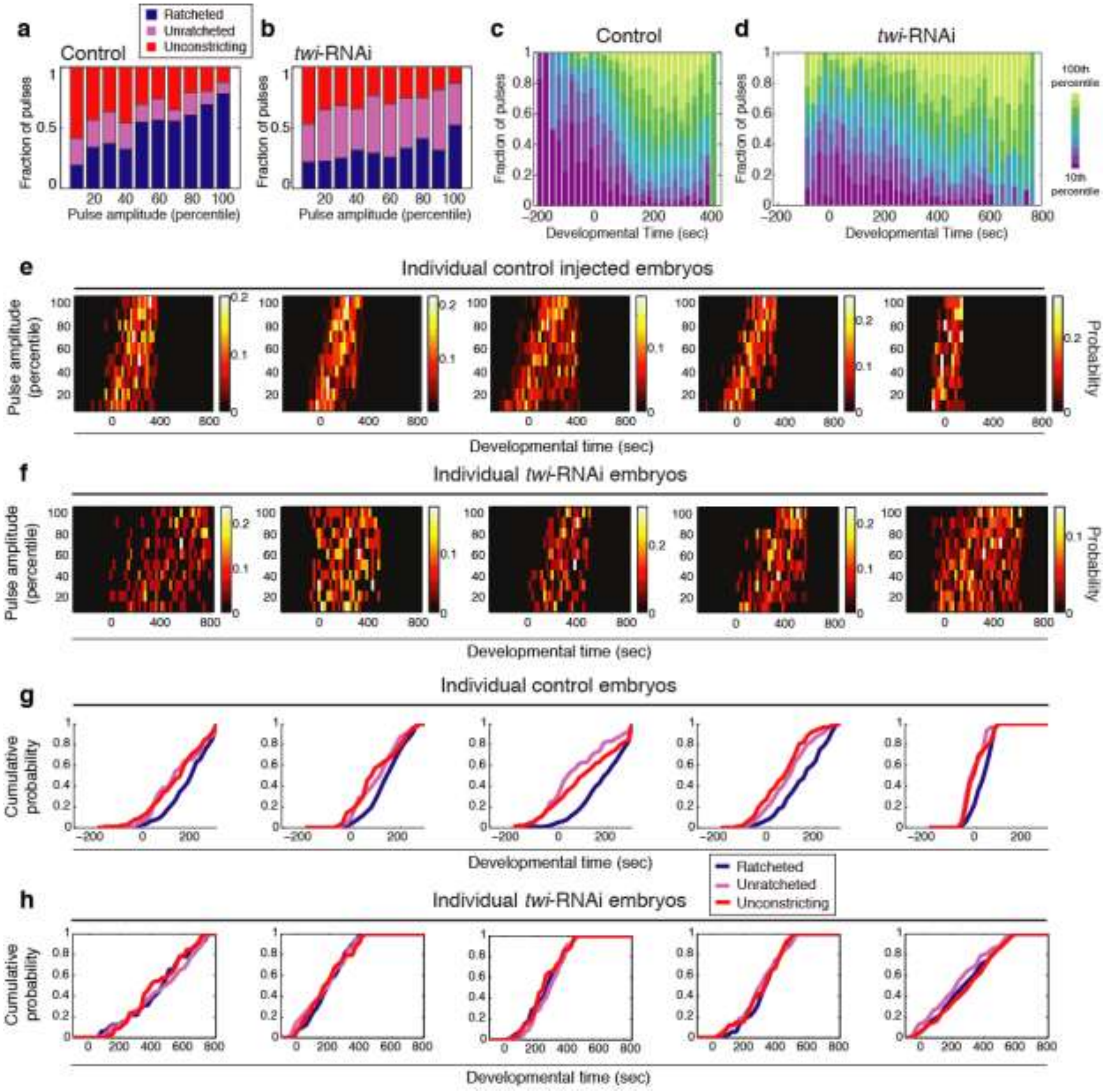

Supplementary Figure 7. **Pulsing dynamics in control injected and *twi*-RNAi tissues**

a-b. The fraction of pulses in each behaviour class shows that *twi*-RNAi pulses across all pulse amplitude-bins exhibit significant non-ratcheted pulses (b).

c-d. The fraction of pulses in each amplitude-bin is shown for each temporal bin in developmental time, highlighting the rapid transition from low-amplitude pulses into high-amplitude pulses in wild-type (c) but not *twi*-RNAi cells (d).

e-f. Probability distributions for the timing of pulses in individual control embryos (e) reveal the temporal progression of low- to high-amplitude pulses. The overlapping probability distributions of different amplitude-bins over time in individual *twi*-RNAi embryos (f) indicate the disruption of this temporal progression.

g-h. Cumulative probability distributions for the timing of pulses in individual control embryos (g) reveal the transition from unratcheted and unconstricting to ratcheted pulses, while the timings of unratcheted and ratcheted pulses in individual *twi*-RNAi embryos overlap (h), showing that the transition is disrupted.

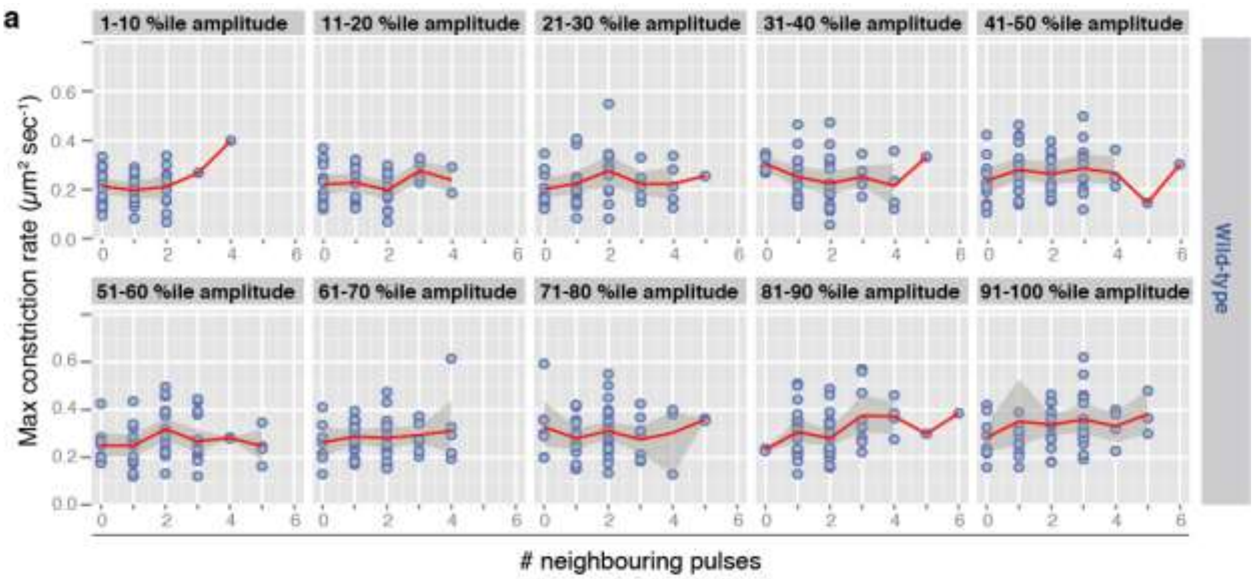

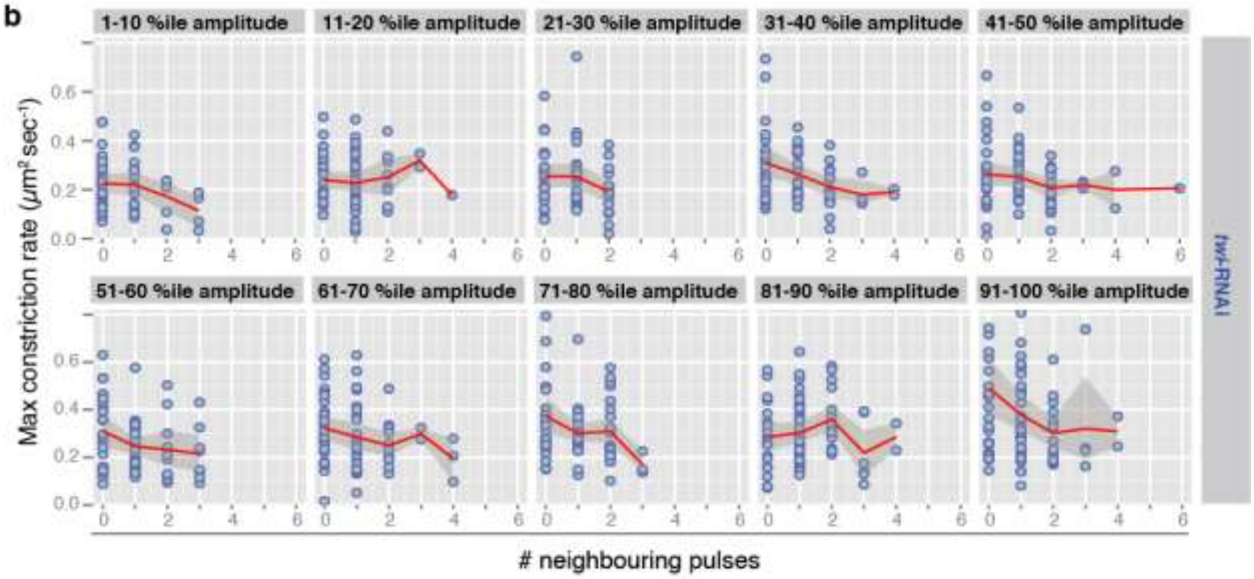

Supplementary Figure 8. **Interactions between neighbouring contractions**

a-b. Wild-type pulses on average interact non-competitively while *twi*-RNAi pulses act competitively. For pulses within single amplitude-bins, the maximum constriction rate attained during a pulse (y-axis) and number of neighbouring pulses (x-axis) were quantified in wild-type (a) and *twi*-RNAi (b) cells. The red lines show the means while shaded areas show standard deviations. Note that the trend line goes up or is flat in wild-type cells, but mostly goes down for *twi*-RNAi cells.

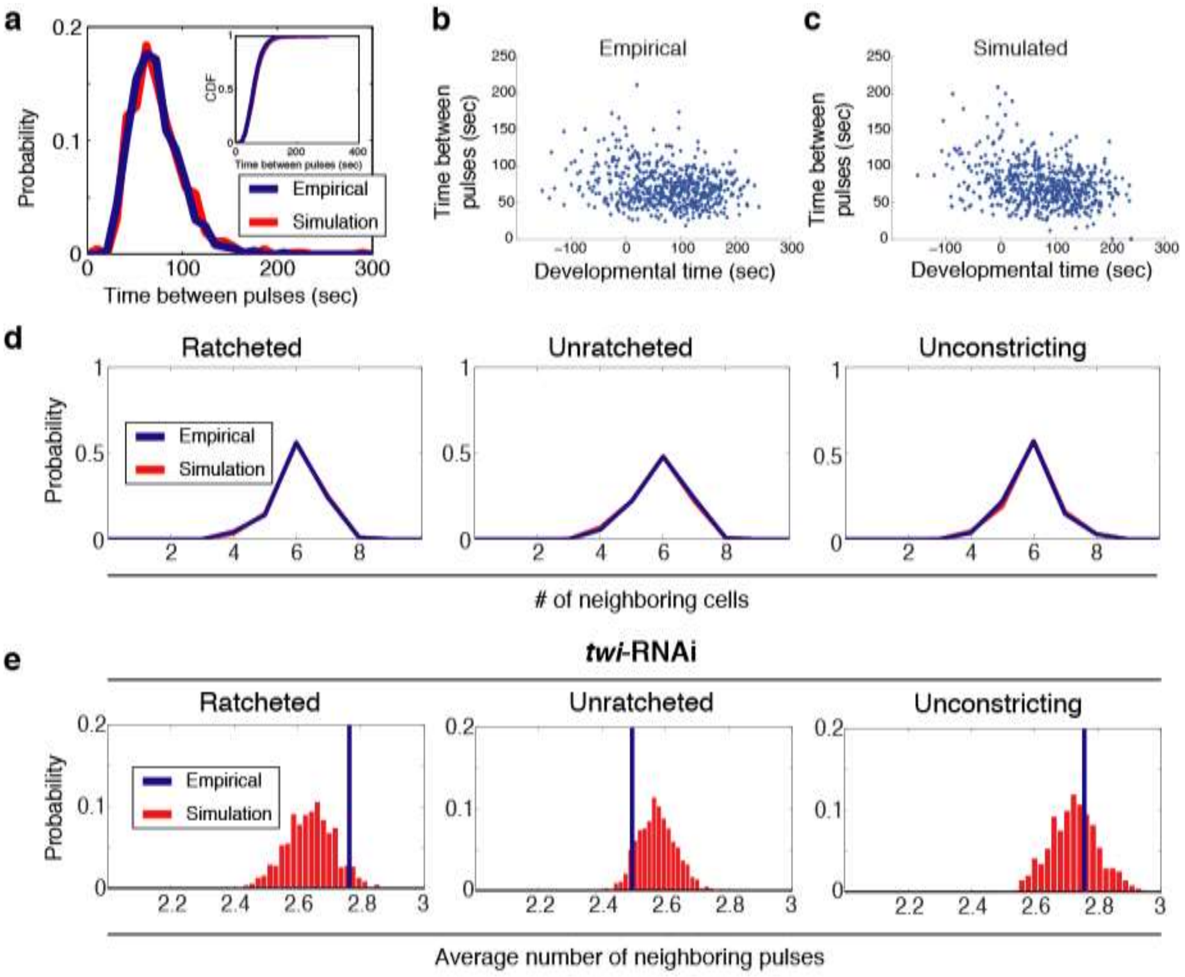

Supplementary Figure 9. **Spatial analysis of pulsing**

a. Pulse spatial randomization procedure preserves pulse periodicity. The probability distribution of time intervals between consecutive pulses for the empirical (blue) and simulated (red) pulsing pattern are very similar ($P > 0.25$, two-sample KS test). Inset: Cumulative distribution function.

b-c. Pulse spatial randomization procedure preserves the temporal dynamics of pulse periodicity. Pulse periodicity is plotted as a function of developmental time for the empirical (b) and simulated (c) pulsing patterns.

d. Pulse spatial randomization procedure preserves the number of cells neighbouring the pulsing cell. The probability distributions of the number of neighbouring cells next to ratcheted, unratcheted, and unconstricting pulses are identical for the empirical (blue) and 1,000 iterations of simulated (red) pulsing patterns.

e. Ratcheted pulses are enriched in neighbouring pulses in *twi*-RNAi cells. The distributions of the average number of neighbouring pulses from spatial randomization simulations (red) and the empirical frequency (blue) with respect to *twi*-RNAi pulses of different area response behaviours.

Supplementary Table 1. **Central pulse timing does not affect analysis of competition.**

**Correlation variables**	**Control variable**	**Partial correlation (P-value)**
Central pulse timing, number of neighbouring pulses	Central pulse amplitude	$R = -0.0201, P > 0.7$
Central pulse timing, maximum constriction rate	Central pulse amplitude	$R = -0.0129, P > 0.8$

Partial Pearson correlation values are shown between the correlation variables while controlling for the specified control variables. All results are shown for wild-type pulses with 51st-100th percentile amplitudes. P-values are against null hypotheses of no correlation.
